# An Inverse Generalized Conversion Filter for State Estimation in Nonlinear Adversarial Sensing Systems

**DOI:** 10.3390/s26103260

**Published:** 2026-05-21

**Authors:** Yi-An Xi, Xin-Hao Dong, Sun-Yong Wu

**Affiliations:** 1 Guangxi Colleges and Universities Key Laboratory of Data Analysis and Computation, School of Mathematics and Computing Science, Guilin University of Electronic Technology, Guilin 541004, China; xya030609@163.com (Y.-A.X.); dxh_7235@163.com (X.-H.D.); 2 Guangxi Key Laboratory of Precision Navigation Technology and Application, Guilin 541004, China; 3 Center for Applied Mathematics of Guangxi, Guilin University of Electronic Technology, Guilin 541004, China

**Keywords:** inverse filtering, generalized conversion-based transformation, nonlinear sensing systems, counter adversarial games, sensor signal processing

## Abstract

In adversarial games involving intelligent sensing systems, inverse filtering plays an important role in the defender’s decision-making by estimating the opponent’s perception based on the defender’s sensor observations. Existing inverse nonlinear filters, such as the inverse quadrature Kalman filter and the inverse extended Kalman filter, are limited in their ability to fully exploit higher-order nonlinear information contained in sensor observations. To address this issue, this paper proposes an inverse generalized-conversion-based filter (I-GCF). Unlike conventional inverse filters, the proposed method not only extracts nonlinear information through deterministic sampling but also constructs a generalized optimal decorrelating transformation function to capture nonlinear observation information that cannot be obtained by the linear minimum mean-square error (LMMSE) estimator. As a result, it enhances the exploitation of higher-order nonlinear sensor information and improves the estimation accuracy and stability of inverse filtering in nonlinear sensing environments. Furthermore, this paper derives general expressions for the time complexities of both GCF and I-GCF, thereby further enriching their theoretical framework. Numerical results demonstrate that, in nonlinear environments, the proposed I-GCF achieves higher estimation accuracy and better stability than conventional inverse filters.

## 1. Introduction

With the widespread application of intelligent sensing systems in complex environments, cognition-related problems in adversarial games have gradually become a research focus [1]. In adversarial games, the opponent observes the state of another system, namely, the defender, in order to make decisions. Conversely, the defender needs to infer the opponent’s observation of itself through its own sensors so as to take countermeasures. For example, in autonomous driving scenarios [2], an opponent vehicle observes and estimates the trajectories of surrounding vehicles through onboard sensors to support its decision-making, whereas a neighboring vehicle acting as the defender performs inverse estimation based on its own sensor observations to infer such interactive behaviors, thereby predicting and avoiding future paths to ensure safer autonomous navigation. In such an adversarial architecture, the opponent system typically employs a forward filter to process sensor measurements and estimate the defender’s state, whereas the defender system uses inverse filtering to infer the opponent’s observation outcomes [3]. Since an inverse filter essentially reconstructs the opponent’s forward estimation process, it is generally not developed independently, but rather derived from the corresponding forward filter. As a result, inverse filters are usually matched with their corresponding forward filters and exhibit theoretical consistency in modeling principles, derivation methods, and performance analysis [4].

In adversarial games under linear environments, the Kalman filter (KF) and the corresponding inverse Kalman filter (I-KF) are optimal for forward filtering estimation [5] and inverse filtering estimation [4], respectively. However, in sensing-system games under nonlinear environments, there is still no theoretically optimal forward estimator [6] or inverse estimator [7]. The extended Kalman filter (EKF) [8] and the corresponding inverse extended Kalman filter (I-EKF) process nonlinear sensor observations within the linear minimum mean-square error (LMMSE) framework by means of local linearization via Taylor-series expansion. Under strongly nonlinear conditions, this strategy leads to significant information loss and, consequently, low estimation accuracy [9]. The unscented Kalman filter (UKF) [10], quadrature Kalman filter (QKF) [11], and cubature Kalman filter (CKF) [12], together with their inverse counterparts, namely, the inverse unscented Kalman filter (I-UKF) [7], inverse quadrature Kalman filter (I-QKF), and inverse cubature Kalman filter (I-CKF) [13], handle nonlinear observations through moment approximation based on deterministic sampling. Although these methods alleviate part of the information loss suffered by EKF and I-EKF and achieve slightly higher estimation accuracy, they still perform nonlinear estimation essentially within the linear affine subspace spanned by the original measurements under the LMMSE framework, and therefore fail to exploit the useful information carried by sensor observations in higher-dimensional spaces [14]. Owing to this underlying limitation, conventional inverse filtering algorithms inevitably face an intrinsic performance bound in nonlinear estimation accuracy. The LMMSE estimation method relies only on first-order and second-order statistical quantities, namely, the mean and covariance, and can only utilize the linear correlation structure between the state and the measurements. For nonlinear systems, the statistical dependence between the state and the measurements is usually reflected not only in first- and second-order statistics, but also in higher-order statistics, such as skewness, kurtosis, and higher-order mixed moments (e.g., third-order and fourth-order moments). However, such higher-order statistical information is often not captured by first- and second-order statistics in the original measurement space. Due to this fundamental limitation, regardless of how the deterministic sampling scheme is modified, conventional inverse filtering algorithms inevitably face a performance bound in nonlinear estimation accuracy caused by the lack of utilization of higher-order information.

To address this issue, this paper aims to develop an inverse filter capable of extracting more nonlinear information from sensor observations and thereby improving estimation accuracy. Inspired by the generalized conversion-based filter (GCF) [6], we propose an inverse generalized-conversion-based filter (I-GCF) with enhanced information extraction capability. The main contributions of this paper are summarized as follows. Unlike conventional methods that merely modify the construction of sampling points, the proposed I-GCF further introduces a generalized optimal conversion-based decorrelation mechanism in addition to deterministic sampling. Specifically, the method constructs a generalized conversion function subject to derivative constraints, which maps the original inverse measurements into a higher-dimensional functional space. In this space, higher-order nonlinear statistical dependencies that cannot be represented by second-order statistics (e.g., covariance) in the original measurement space are re-expressed as second-order correlations between the state and the transformed measurements. Since the I-GCF still adopts the LMMSE estimation structure, this transformation enables the previously unexploitable nonlinear information to be indirectly utilized through the new covariance structure, thereby effectively improving estimation accuracy under nonlinear observation conditions. As a result, in nonlinear inverse estimation problems, the I-GCF achieves lower average RMSE, smaller error peaks, and smoother error variations compared with the I-UKF, I-CKF, and I-QKF. The main contributions of this paper are summarized as follows.

1. *A complete theoretical framework for I-GCF:* To improve the capability of inverse filters in exploiting nonlinear information, this paper develops I-GCF on the basis of the forward GCF and establishes an adversarial framework centered on a generalized conversion-based decorrelating transformation. The proposed I-GCF is used by the defender to infer the estimation results generated when the opponent deploys a forward GCF. Within this adversarial framework, it is assumed that the defender knows all parameters of the opponent’s dynamic system. In the I-GCF algorithm, the defender can further extract, through the inverse generalized conversion-based decorrelating transformation function, the uncorrelated information in its own sensor observations that cannot be obtained through deterministic sampling alone, thereby enhancing the utilization of nonlinear information. Numerical experiments further verify the superior estimation performance and strong robustness of I-GCF under strongly nonlinear conditions. Different from conventional formulations, this paper systematically introduces the generalized conversion mechanism of the forward GCF into the inverse filtering problem for the first time, and constructs a generalized optimal conversion-based decorrelation mechanism tailored for adversarial perception scenarios within the inverse estimation framework. This mechanism enables inverse filtering to go beyond the conventional LMMSE-based formulation and to further extract effective decorrelated information induced by the original inverse observations within a constrained functional space. Moreover, the I-GCF is capable of extracting high-dimensional nonlinear information beyond the LMMSE subspace that cannot be obtained solely through deterministic sampling, thereby further enhancing the utilization of nonlinear observation information. Extensive numerical experiments further demonstrate that the I-GCF achieves superior estimation performance and strong robustness under strongly nonlinear conditions. In particular, in the FM demodulation system, the average RMSE is reduced by approximately 5%, while in the three-dimensional Lorenz chaotic system, the I-GCF consistently maintains the lowest RMSE in the main error growth interval. At peak error, it achieves a reduction of approximately 20% compared with I-UKF and I-CKF, and nearly one order of magnitude compared with I-EKF. Moreover, it maintains the lowest error level under both matched and mismatched model conditions, demonstrating stronger robustness and generalization capability.
2. *Time complexities of GCF and I-GCF:* To clarify the computational cost incurred by the higher estimation accuracy of GCF and I-GCF, and to facilitate the selection of suitable practical adversarial scenarios for their deployment, this paper derives the time complexities of both methods. Since the deterministic sampling strategies adopted by GCF and I-GCF are not fixed, the derived results are presented in the form of parameterized general expressions, where the parameter corresponds to the time complexity of the deterministic sampling component.
3. *Mismatched forward filters:* Conventional inverse filters usually rely on the assumption that the opponent employs the corresponding forward filter. In practical adversarial systems, however, the forward filter actually deployed by the opponent may not be consistent with the assumption adopted by the inverse filter. Numerical results in this paper show that, even when the opponent uses a mismatched forward filter, I-GCF can still maintain robust estimation accuracy and stability.

The remainder of this paper is organized as follows. Section 2 presents the system model in the nonlinear adversarial framework. Section 3 reviews the algorithmic procedure of the forward GCF and the derivation of the forward generalized conversion-based decorrelating function. Section 4 derives the complete algorithmic procedure of I-GCF and the inverse generalized conversion-based decorrelating function on the basis of the theory of the forward GCF. Section 5 presents the derivation and results of the time complexity analysis for the forward GCF and I-GCF, thereby further completing the overall theoretical framework. Section 6 presents numerical results, where metrics such as RMSE are used to quantitatively demonstrate that I-GCF achieves better estimation accuracy and stability than existing mainstream inverse filters under both theoretically matched forward–inverse filtering scenarios and practical mismatched scenarios. Finally, Section 7 concludes this paper.

**Remark 1.**  *All experiments in this study were conducted using MATLAB (v2024).*

## 2. System Model

In practical adversarial sensing scenarios, typical tasks generally fall into two categories. The first category is maneuvering-target perception scenarios [15], such as target tracking, autonomous driving interaction, and radar/sonar detection. In such problems, the defender’s state often exhibits complex maneuverability and nonlinear dynamic characteristics, while the opponent observes it through range, bearing, or other nonlinear sensors, and further forms state estimates to support subsequent decision-making [16]. The second category is signal reconnaissance scenarios [17], such as electronic reconnaissance, communication interception, and SDR perception. In such problems, the relationships between system states, phase, frequency offset, and sensor outputs also usually exhibit significant nonlinearity [18]. Based on these two representative practical backgrounds, this paper adopts nonlinear system models to provide an abstract representation of real adversarial sensing systems. Furthermore, the Lorenz model and the FM model selected in Section 6 correspond to typical nonlinear adversarial sensing tasks in maneuvering-target perception and signal reconnaissance, respectively.

In the abstracted nonlinear system, the state evolution of the defender can be modeled as a discrete-time stochastic system with the Markov property, denoted by ${\left\{x_{k}\right\}}_{k\geq 0}$. Here, $x_{k}\in R^{n_{x}\times 1}$ represents the defender’s state vector at time step *k*, which is fully transparent and known to the defender itself. Owing to the Markov property of the system, when the time step evolves from *k* to k+1, the defender state $x_{k+1}$ depends only on $x_{k}$ and is independent of any state prior to time step *k*. The state evolution model from time step *k* to k+1 can be written as

(1) $$x_{k+1}=f\left(x_{k}\right)+w_{k}$$

where f(·) is the state transition function, and $w_{k}∼N\left(0_{n_{x}\times 1},G\right)$ denotes the process noise, which is the main source of uncertainty in the state transition process, with covariance matrix $G\in R^{n_{x}\times n_{x}}$.

Meanwhile, the opponent relies on its deployed sensors, such as radar, sonar, or vision sensors, to observe the defender’s state. At time step *k*, the defender state observed by the opponent is denoted by $z_{k}\in R^{n_{z}\times 1}$, and the corresponding observation model is given by

(2) $$z_{k}=h\left(x_{k}\right)+v_{k}$$

where the observation noise $v_{k}∼N\left(0_{n_{z}\times 1},H\right)$ arises from sensor thermal noise, quantization error, or environmental interference, with covariance matrix $H\in R^{n_{z}\times n_{z}}$. The observation function h(·) represents the specific nonlinear sensing mechanism, such as target ranging or bearing measurement, that maps the true defender state $x_{k}$ to the sensor measurement. The opponent processes the historical sensor measurement sequence ${\left\{z_{i}\right\}}_{i=1}^{k}$ through a forward filter to obtain the posterior estimate of the defender’s state, denoted by ${\hat{x}}_{k}$, and then takes corresponding actions according to this posterior estimate and its own game strategy. At the same time, the defender also observes the opponent’s state through its own deployed sensors.

At time step *k*, the defender observes the opponent’s actions through its sensors, and the resulting observation is denoted by $a_{k}\in R^{n_{a}\times 1}$. The corresponding observation model is expressed as

(3) $$a_{k}=t\left({\hat{x}}_{k}\right)+\epsilon_{k}$$

where t(·) is a composite function that includes both the mapping from the opponent’s strategy to its action and the mapping from the defender’s sensor observation process. The additive noise $\epsilon_{k}∼N\left(0_{n_{a}\times 1},\Sigma_{\epsilon }\right)$ represents the measurement error introduced when the defender observes the opponent through its sensors, with covariance matrix $\Sigma_{\epsilon }\in R^{n_{a}\times n_{a}}$.

The main objective of the defender is to estimate the opponent’s behavior based on its own sensor observations so as to predict, prevent, or counteract the opponent’s actions. This task is typically accomplished by deploying an inverse filter to estimate the opponent’s estimate of the defender’s state, namely, ${\hat{x}}_{k}$. The resulting inverse estimate is denoted by ${\hat{\hat{x}}}_{k}$. When the state transition function f(·), the opponent’s sensor observation function h(·), and the defender’s sensor observation function t(·) all satisfy linear assumptions, the inverse Kalman filter (I-KF) achieves optimal inverse estimation. However, real physical systems are usually much more complex, and in most cases, the sensing mappings are nonlinear or even implicit functions. Under such conditions, the estimation accuracy of I-KF deteriorates sharply and may even diverge [9]. To date, for the inverse filtering problem arising in such nonlinear dynamic systems, no universal inverse filter with theoretical optimality has been developed. Therefore, this paper proposes a new inverse filter suitable for nonlinear environments. Compared with existing mainstream inverse filters, the proposed filter can achieve higher estimation accuracy. Its detailed architecture is presented in Section 4. In this paper, $w_{k}$, $v_{k}$, and $\epsilon_{k}$ are regarded as temporally independent and identically distributed random sequences, and it is assumed by default that both players in the game, namely, the defender and the opponent, know all functions and noise parameters in the system.

## 3. Forward GCF

The forward generalized-conversion-based filter (GCF) constructs an optimal transformation through functional optimization, thereby avoiding the explicit construction of the optimal decorrelating transformation function. By using deterministic sampling schemes, such as the unscented transform (UT) [19], Gauss–Hermite quadrature (GHQ) [20], and spherical–radial cubature rules [12], the forward GCF projects nonlinear observation information into a null space, thereby extracting all uncorrelated observation information obtainable under the imposed constraints and using it for state estimation [6].

The forward GCF performs recursive estimation based on the dynamic system in (1) and (2). In the deterministic sampling stage, various sampling schemes, such as the unscented transform (UT) and Gauss–Hermite quadrature (GHQ), can be adopted. In the following, the algorithm is presented by taking the GHQ sampling as an example. Let $\xi_{i}$ denote the obtained standard sampling points, $\omega_{i}$ the corresponding weights, and *m* the number of sampling points, i.e., 1≤i≤m. Let ${\left\{s_{i,k}^{*}\right\}}_{1\leq i\leq mn_{x}}$ denote the time-propagated sampling points, and let ${\left\{q_{i,k|k-1}\right\}}_{1\leq i\leq mn_{x}}$ denote the measurement sampling points.

*Time update:*

(4) $$\begin{array}{c} \;\begin{array}{cc} & s_{i,k-1}=\xi_{i}\sqrt{P_{k-1}}+{\hat{x}}_{k-1},\;\forall i=1,\ldots ,mn_{x} \end{array} \end{array}$$

(5) $$\begin{array}{c} \;\begin{array}{cc} & s_{i,k|k-1}^{*}=f\left(s_{i,k-1}\right),\;\forall i=1,\ldots ,mn_{x} \end{array} \end{array}$$

(6) $$\begin{array}{c} {\hat{x}}_{k|k-1}=\sum_{i=1}^{mn_{x}}\omega_{i}s_{i,k|k-1}^{*}\\ P_{k|k-1}=\sum_{i=1}^{mn_{x}}\omega_{i}s_{i,k|k-1}^{*}{\left(s_{i,k|k-1}^{*}\right)}^{T}-{\hat{x}}_{k|k-1}{\hat{x}}_{k|k-1}^{T}+G \end{array}$$

(7) $$\begin{array}{c} \;\begin{array}{cc} & X_{k}=\left[s_{1,k|k-1}^{*},s_{2,k|k-1}^{*},\ldots ,s_{mn_{x},k|k-1}^{*}\right] \end{array} \end{array}$$

(8) $$\begin{array}{c} \;\begin{array}{cc} & U={\left[\omega_{1},\omega_{2},\ldots ,\omega_{mn_{x}}\right]}^{T} \end{array} \end{array}$$

*Measurement update:*

(9) $$\begin{array}{c} \;\begin{array}{cc} & q_{i,k|k-1}=h\left(s_{i,k|k-1}^{*}\right),\;\forall i=1,\ldots ,mn_{x} \end{array} \end{array}$$

(10) $$\begin{array}{c} \;\begin{array}{cc} & {\hat{z}}_{k|k-1}=\sum_{i=1}^{mn_{x}}\omega_{i}q_{i,k|k-1} \end{array} \end{array}$$

(11) $$\begin{array}{c} Z_{k}=\left[q_{1,k|k-1},q_{2,k|k-1},\ldots ,q_{mn_{x},k|k-1}\right]\\ Z_{i,k}^{a}=e_{i,n_{z}}\left[Z_{k},z_{k}^{a}\right] \end{array}$$

(12) $$\begin{array}{c} \;\begin{array}{cc} & M_{x,k}=\left(X_{k}-{\hat{x}}_{k|k-1}1_{1\times mn_{x}}\right)\mathrm{diag}\left(U\right) \end{array} \end{array}$$

(13) $$\begin{array}{c} \;\begin{array}{cc} & M_{y}=\sum_{i=1}^{mn_{x}}\omega_{i}\left(e_{i,mn_{x}}-U\right){\left(e_{i,mn_{x}}-U\right)}^{T} \end{array} \end{array}$$

(14) $$\begin{array}{c} \;\begin{array}{cc} & C_{i,k}^{T}=\left[Q_{0,i,k},Q_{i,k}\right]\left[\begin{array}{c} R_{0,i,k}\\ 0 \end{array}\right], \end{array} \end{array}$$

(15) $$\begin{array}{c} \;\begin{array}{cc} & Q_{k}={\left[Q_{1,k},Q_{2,k},\ldots ,Q_{n_{z},k}\right]}^{T} \end{array} \end{array}$$

(16) $$\begin{array}{c} \;\begin{array}{cc} & {\hat{x}}_{k}={\hat{x}}_{k|k-1}-M_{x,k}Q_{k}^{T}{\left(Q_{k}M_{y}Q_{k}^{T}\right)}^{-1}Q_{k}U \end{array} \end{array}$$

(17) $$\begin{array}{c} \;\begin{array}{cc} & P_{k}=P_{k|k-1}-M_{x,k}Q_{k}^{T}{\left(Q_{k}M_{y}Q_{k}^{T}\right)}^{-1}Q_{k}M_{x,k}^{T} \end{array} \end{array}$$

where the weights satisfy $\omega_{i}=1/\left(\pi^{n_{x}/2}\right)$, and $e_{i,mn_{x}}$ denotes the *i*th standard basis vector of dimension $mn_{x}$. For $C_{i,k}$, each dimension corresponds to the first $mn_{x}$ columns of the numerical-derivative constraint matrix constructed from the measurement component $Z_{i,k}^{a}$. Moreover, (15) is obtained by QR decomposition, and the column vectors of $Q_{j,k}$ form an orthogonal basis for the null space of $C_{j,k}$.

**Remark 2**  (Procedure for obtaining the standard sampling points $\xi_{i}$ by GHQ)**.** *Under the one-dimensional weight function $e^{-x^{2}}$, all zeros of the Nth-order Hermite polynomial are selected as the sampling points, and the corresponding one-dimensional weights are calculated. For an d-dimensional variable, the multidimensional sampling points are constructed by the tensor-product extension of the one-dimensional sampling points, resulting in a total of $N^{d}$ sampling points. The weight of each multidimensional sampling point is obtained as the product of the corresponding one-dimensional weights in each dimension, and is uniformly multiplied by the normalization factor $1/\pi^{d/2}$.*

## 4. I-GCF

**Figure 1:**
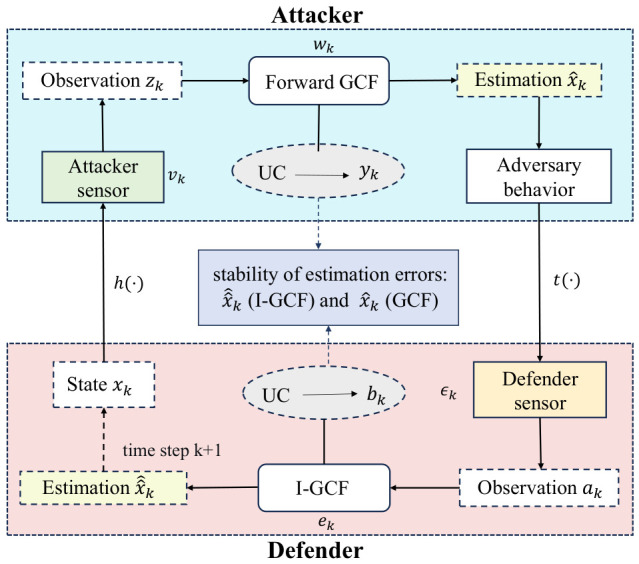
Adversarial flowchart of GCF and I-GCF.

The GCF, by means of generalized conversion-based techniques, can effectively extract the uncorrelated information in observations that is difficult to be directly utilized by the LMMSE estimator in complex nonlinear systems, thereby achieving high state-estimation accuracy in forward nonlinear filtering [6]. In adversarial environments, both players usually perform state-cognition estimation simultaneously. On the one hand, the opponent employs a forward filter to estimate the defender’s state; on the other hand, the defender needs to use an inverse filter to infer the opponent’s cognitive process, i.e., to estimate the opponent’s estimate of the defender’s state obtained through the forward filter. However, existing inverse filters, such as the I-EKF and I-UKF, often suffer from limited capability in extracting nonlinear information when dealing with strongly nonlinear systems, resulting in unsatisfactory estimation accuracy. To address this issue, this paper incorporates the advantage of GCF in nonlinear information extraction into the inverse filtering framework and constructs an inverse generalized-conversion-based filter (I-GCF), so as to improve the defender’s cognitive inference capability in complex nonlinear environments and thereby enhance its situational awareness and adversarial decision-making ability. Figure 1 illustrates the adversarial process in which both players perform the game using GCF/I-GCF.

### 4.1. Generalized Optimal Conversion Function

In nonlinear estimation theory, conventional inverse filtering methods are generally constrained by the update structure of the LMMSE framework. Since the LMMSE estimator essentially performs orthogonal projection within the linear affine subspace spanned by the original measurements, its solution space can only exploit the first- and second-order statistics of the variables, and therefore the higher-order nonlinear information contained in the observations cannot be utilized [14]. In contrast, the optimal conversion function can project the original observations into a new feature space through a highly nonlinear mapping mechanism, thereby changing the representation of the original observation information and transforming part of the higher-order statistical information, which cannot be resolved by the LMMSE estimator, into equivalent measurements that can be directly processed within the LMMSE update framework. Since solving for an optimal conversion function with a specific analytical form in complex systems is essentially an extremely difficult functional optimization problem, this paper constructs an inverse generalized optimal conversion function according to its theoretical requirements.

In the I-GCF, the composite observation variable $a_{k}$ obtained in (3) is denoted in the inverse generalized optimal conversion function part by a, and the observation variable obtained from sensor measurements is denoted by $a^{a}$. The construction of the generalized optimal conversion function in I-GCF starts from a continuous decorrelating transformation function, and then discretizes it into a generalized function through functional optimization.

The continuous decorrelating transformation function is defined as

(18) $$b=\bar{g}\left(a\right),$$

where b denotes the inversely transformed decorrelated observation, and $\bar{g}\left(\cdot \right)$ denotes the continuous optimal decorrelating transformation function. Under the LMMSE framework, $\bar{g}\left(\cdot \right)$ can be expressed as

(19) $$\hat{\bar{g}}\left(\cdot \right)=\arg\max_{\bar{g}\left(\cdot \right)}{\bar{P}}_{xb}\;{\bar{P}}_{b}^{-1}\;{\bar{P}}_{xb}^{T},$$

where ${\bar{P}}_{xb}$ denotes the cross-covariance matrix between the state variable $\hat{x}$ and the decorrelated observation b, and ${\bar{P}}_{b}$ denotes the covariance matrix of the decorrelated observation b. After deterministic sampling, let $\hat{X}=\left[{\hat{x}}^{1},\ldots ,{\hat{x}}^{n_{s}}\right]$ be the state-sample matrix, $A=\left[a^{1},\ldots ,a^{n_{s}}\right]$ be the observation-sample matrix, $B=\left[b^{1},\ldots ,b^{n_{s}}\right]$ be the sample matrix of decorrelated transformed points corresponding to A, and $\bar{U}={\left[{\bar{w}}_{1},\ldots ,{\bar{w}}_{n_{s}}\right]}^{T}$ be the weight matrix of the sampling points. Then, the above statistics can be written as

(20) $$\hat{b}=B\bar{U},$$

(21) $${\bar{P}}_{xb}={\bar{M}}_{x}B^{T},$$

(22) $${\bar{P}}_{b}=B{\bar{M}}_{b}B^{T},$$

where ${\bar{M}}_{x}=\left(\hat{X}-\hat{\hat{\bar{x}}}1_{1\times n_{s}}\right)\mathrm{diag}\left(\bar{U}\right)$, and ${\bar{M}}_{b}=\sum_{s=1}^{n_{s}}{\bar{w}}_{s}\left(e_{s,n_{s}}-\bar{U}\right){\left(e_{s,n_{s}}-\bar{U}\right)}^{T}$. Here, $\hat{X}$ is the state-sample matrix of the sampling points. Then, (19) can be discretized into the following optimization problem with respect to the sample matrix B:

(23) $$\max_{B}{\bar{M}}_{x}B^{T}{\left(B{\bar{M}}_{b}B^{T}\right)}^{-1}B{\bar{M}}_{x}^{T}.$$

To solve this optimization problem, it is necessary to restrict the underlying space of the problem to a solvable finite-dimensional space so as to suppress the higher-order approximation errors caused by the aforementioned deterministic sampling. Therefore, numerical-derivative constraints need to be imposed on the optimization problem in (23). By constructing the augmented sample row vector corresponding to the *i*th component of the observation vector a as

(24) $$A_{i}^{a}=e_{i,n_{a}}\left[A,a^{a}\right],$$

and then computing the numerical differentiation matrix ${\bar{C}}_{i}^{\left(n\right)}$ of the augmented row vector, the constraint matrix ${\bar{C}}_{i}$ can be obtained by taking its first $n_{s}$ rows. The constraint on the *i*th block $B_{0,i}^{T}$ of the transformed sample matrix can then be expressed as

(25) $${\bar{C}}_{i}B_{0,i}^{T}=0.$$

By performing QR decomposition on the *i*th constraint matrix ${\bar{C}}_{i}$, an orthogonal basis for the null space of the constraint matrix can be obtained. The QR decomposition is given by

(26) $${\bar{C}}_{i}^{T}=\left[\begin{array}{cc} {\bar{Q}}_{0,i} & {\bar{Q}}_{i} \end{array}\right]\left[\begin{array}{c} {\bar{R}}_{0,i}\\ 0 \end{array}\right].$$

Here, $B_{0,i}^{T}\in \mathrm{Null}\left({\bar{C}}_{i}\right)$. Therefore, the *i*th block of the transformed sample matrix satisfying the constraint can be written as

(27) $$B_{0,i}^{T}={\bar{Q}}_{i}{\bar{W}}_{i},$$

where ${\bar{W}}_{i}$ is an arbitrary full-rank matrix. By concatenating all ${\bar{Q}}_{i}$, the orthogonal basis of the overall constrained space can be obtained as

(28) $$\bar{Q}={\left[{\bar{Q}}_{1},\ldots ,{\bar{Q}}_{n_{a}}\right]}^{T}.$$

According to the property that all linearly feasible solutions can be represented by the orthogonal basis of the null space [21], all solutions of (23) can be expressed by $\bar{Q}$ as

(29) $$B=\bar{W}\cdot \bar{Q}+\mathrm{diag}\left(b^{a}\right)1.$$

where $b^{a}$ is the decorrelated transformed value of the true sensor observation $a^{a}$. Substituting this general solution into (20)–(22) yields

(30) $${\bar{P}}_{xb}={\bar{M}}_{x}{\bar{Q}}^{T}{\bar{W}}^{T},\;{\bar{P}}_{b}=\bar{W}\cdot \bar{Q}\cdot {\bar{M}}_{b}{\bar{Q}}^{T}{\bar{W}}^{T}.$$

Accordingly, the inverse update gain can be obtained as

(31) $$\bar{K}={\bar{M}}_{x}{\bar{Q}}^{T}{\left(\bar{Q}\cdot {\bar{M}}_{b}{\bar{Q}}^{T}\right)}^{-1}\bar{Q}.$$

**Remark 3.**  *Representing the optimal conversion function as the general solution in the form of a linear combination of null-space basis vectors, and using it to represent the decorrelated information, essentially bypasses the actual solution of the optimal decorrelating transformation function through subspace projection, thereby accomplishing the extraction of decorrelated information. In this way, the originally intractable functional optimization problem is transformed into a matrix optimization problem in the null-space subspace.*

### 4.2. I-GCF Algorithm

As a technical tool for estimating the estimation result of the forward filter, the inverse filter does not derive its dynamic state transition equation from physical logic; rather, it is obtained according to the algorithmic logic of the corresponding forward filter. It is worth noting that, due to the specific structure of the forward GCF, its state update equation in (16) differs from those of conventional forward filters, such as KF and UKF, in that it does not explicitly contain the actual observation of the opponent’s sensor, $z_{k}^{a}$. Therefore, unlike conventional inverse filters, one cannot directly replace the opponent sensor’s actual observation $z_{k}$ with the observation function in (2) and recursively simplify the result to obtain a state transition equation between ${\hat{x}}_{k}$ and ${\hat{x}}_{k-1}$ [3]. However, parameters such as $M_{x,k}$ and $Q_{k}$ in (16) still depend on ${\hat{x}}_{k-1}$ and the opponent sensor’s actual observation $z_{k}^{a}$ during sample generation and statistical computation. Since the defender cannot directly access the opponent’s sensor measurements, it can only generate simulated observations by substituting its own true state $x_{k}$ and the random process noise $v_{k}$ into (2). Therefore, for I-GCF, the generation of ${\hat{x}}_{k}$ can be regarded as the output of a single simulated forward GCF update under the condition that ${\hat{x}}_{k-1}$ and $x_{k}$ are given. This complete generation process can be viewed as an abstract inverse state transition equation.

Due to the high complexity of this process, this paper represents the inverse state transition equation by the following implicit function:

(32) $${\hat{x}}_{k}=\tilde{f}\left({\hat{x}}_{k-1},P_{k-1}^{\prime },x_{k},v_{k}\right)$$

where $P_{k-1}^{\prime }$ is an approximation of the forward covariance $P_{k-1}$, which is simulated from ${\hat{x}}_{k-1}$ and $x_{k}$. For convenience in subsequent calculations and for theoretical clarity of the algorithm, it is treated as an exogenous variable and iterated separately. The variable $v_{k}$ participates in the generation of simulated observations and is involved in the corresponding computations during the simulated execution of a single forward GCF update. Owing to its randomness, it is also treated as an exogenous variable in this paper. Considering that $v_{k}$ has a complicated and nonadditive effect on ${\hat{x}}_{k}$ in the simulated GCF update, this paper constructs the augmented vector $r_{k}={\left[{\hat{x}}_{k-1}^{T},v_{k}^{T}\right]}^{T}$ ($n_{r}=n_{x}+n_{v}$) to handle this effect. Then, the inverse state transition equation can be rewritten as

(33) $${\hat{x}}_{k}=\tilde{f}\left(r_{k-1},P_{k-1}^{\prime },x_{k}\right).$$

Table 1 presents the complete pseudocode procedure of the implicit function.

The I-GCF performs recursive estimation based on the dynamic system in (33) and (3). I-GCF can likewise adopt various sampling schemes. Here, GHQ sampling is still taken as an example. Considering the augmentation in (33), the estimated state and covariance matrix used in sampling are defined as

(34) $${\hat{r}}_{k}={\left[\begin{array}{cc} {\hat{\hat{x}}}_{k}^{T} & 0_{1\times n_{v}} \end{array}\right]}^{T},\;{\bar{P}}_{k}^{r}=\left[\begin{array}{cc} {\bar{P}}_{k} & 0_{n_{x}\times n_{v}}\\ 0_{n_{v}\times n_{x}} & H \end{array}\right].$$

where ${\bar{P}}_{k}$ is the inverse estimation covariance. ${\bar{P}}_{0}^{r}$ is constructed in an augmented form using ${\bar{P}}_{0}$ and H, where H denotes the forward sensor observation noise covariance, and ${\bar{P}}_{0}$, as the initial inverse covariance, is generally specified directly in practical systems. The specific recursive procedure is as follows.

*Time update:*

(35) $$\begin{array}{c} \;\begin{array}{cc} & {\bar{s}}_{j,k-1}=\xi_{j}\sqrt{{\bar{P}}_{k-1}^{r}}+{\hat{r}}_{k-1},\;\forall j=1,\ldots ,mn_{r} \end{array} \end{array}$$

(36) $$\begin{array}{c} {\bar{s}}_{j,k|k-1}^{*}=f\left({\bar{s}}_{j,k-1},P^{\prime },x_{k}\right),\;\forall j=1,\ldots ,mn_{r}\\ {\hat{x}}_{j,k|k-1}^{s}=\Pi_{x}{\bar{s}}_{j,k|k-1}^{*}\\ v_{j,k|k-1}^{s}=\Pi_{v}{\bar{s}}_{j,k|k-1}^{*} \end{array}$$

(37) $$\begin{array}{c} {\hat{\hat{x}}}_{k|k-1}=\sum_{j=1}^{mn_{x}}{\bar{\omega }}_{j}{\hat{x}}_{j,k|k-1}^{s}\\ {\bar{P}}_{k|k-1}=\sum_{j=1}^{mn_{x}}{\bar{\omega }}_{j}{\hat{x}}_{j,k|k-1}^{s}{\left({\hat{x}}_{j,k|k-1}^{s}\right)}^{T}-{\hat{\hat{x}}}_{k|k-1}{\hat{\hat{x}}}_{k|k-1}^{T} \end{array}$$

(38) $$\begin{array}{c} \;\begin{array}{cc} & {\hat{X}}_{k}=\left[{\hat{x}}_{1,k|k-1}^{s},{\hat{x}}_{2,k|k-1}^{s},\ldots ,{\hat{x}}_{mn_{r},k|k-1}^{s}\right] \end{array} \end{array}$$

(39) $$\begin{array}{c} \;\begin{array}{cc} & \bar{U}={\left[{\bar{\omega }}_{1},{\bar{\omega }}_{2},\ldots ,{\bar{\omega }}_{mn_{r}}\right]}^{T} \end{array} \end{array}$$

where the weights satisfy ${\bar{\omega }}_{j}=1/\left(\pi^{n_{x}/2}\right)$, and $\xi_{j}$ denotes the standard sampling points obtained by GHQ sampling. If other deterministic sampling schemes are adopted, the I-GCF differs only in the values and the number of these standard sampling points. The vectors ${\bar{s}}_{i,k-1}$ are the transformed sampling points generated around the center point ${\hat{r}}_{k}$. This construction is intended to reduce the impact of changing the sampling scheme on the algorithm, enhance algorithmic stability, and reduce the time complexity caused by repeated sampling. The projection matrices

$$\Pi_{x}=\left[\begin{array}{c} I_{n_{x}},0_{n_{x}n_{v}} \end{array}\right]\;\mathrm{and}\;\Pi_{v}=\left[\begin{array}{c} 0_{n_{v}n_{x}},I_{n_{v}} \end{array}\right]$$

are used to extract the state dimension and the noise dimension of the augmented estimated variable for the subsequent optimal conversion.

*Measurement update:*

(40) $$\begin{array}{c} \;\begin{array}{cc} & {\bar{q}}_{j,k|k-1}=h\left({\hat{x}}_{j,k|k-1}^{s}\right)+v_{j,k|k-1}^{s},\;\forall j=1,\ldots ,mn_{r} \end{array} \end{array}$$

(41) $$\begin{array}{c} \;\begin{array}{cc} & {\hat{a}}_{k|k-1}=\sum_{j=1}^{mn_{x}}{\bar{\omega }}_{j}{\bar{q}}_{j,k|k-1} \end{array} \end{array}$$

(42) $$\begin{array}{c} A_{k}=\left[{\bar{q}}_{1,k|k-1},{\bar{q}}_{2,k|k-1},\ldots ,{\bar{q}}_{mn_{r},k|k-1}\right]\\ A_{j,k}^{a}=e_{j,n_{a}}\left[A_{k},a_{k}^{a}\right] \end{array}$$

(43) $$\begin{array}{c} \;\begin{array}{cc} & {\bar{M}}_{x,k}=\left({\hat{X}}_{k}-{\hat{\hat{x}}}_{k|k-1}1_{1\times mn_{r}}\right)\mathrm{diag}\left(\bar{U}\right) \end{array} \end{array}$$

(44) $$\begin{array}{c} \;\begin{array}{cc} & {\bar{M}}_{y}=\sum_{j=1}^{mn_{x}}{\bar{\omega }}_{j}\left(e_{j,mn_{r}}-\bar{U}\right){\left(e_{j,mn_{r}}-\bar{U}\right)}^{T} \end{array} \end{array}$$

(45) $$\begin{array}{c} \;\begin{array}{cc} & {\bar{C}}_{j,k}^{T}=\left[{\bar{Q}}_{0,j,k},{\bar{Q}}_{j,k}\right]\left[\begin{array}{c} {\bar{R}}_{0,j,k}\\ 0 \end{array}\right], \end{array} \end{array}$$

(46) $$\begin{array}{c} \;\begin{array}{cc} & {\bar{Q}}_{k}={\left[{\bar{Q}}_{1,k},{\bar{Q}}_{2,k},\ldots ,{\bar{Q}}_{n_{a},k}\right]}^{T} \end{array} \end{array}$$

(47) $$\begin{array}{c} \;\begin{array}{cc} & {\hat{\hat{x}}}_{k}={\hat{\hat{x}}}_{k|k-1}-{\bar{M}}_{x,k}{\bar{Q}}_{k}^{T}{\left({\bar{Q}}_{k}{\bar{M}}_{y}{\bar{Q}}_{k}^{T}\right)}^{-1}{\bar{Q}}_{k}\bar{U} \end{array} \end{array}$$

(48) $$\begin{array}{c} \;\begin{array}{cc} & {\bar{P}}_{k}={\bar{P}}_{k|k-1}-{\bar{M}}_{x,k}{\bar{Q}}_{k}^{T}{\left({\bar{Q}}_{k}{\bar{M}}_{y}{\bar{Q}}_{k}^{T}\right)}^{-1}{\bar{Q}}_{k}{\bar{M}}_{x,k}^{T} \end{array} \end{array}$$

(49) $$\begin{array}{c} \;\begin{array}{cc} & {\bar{P}}_{k}^{r}=\left[\begin{array}{cc} {\bar{P}}_{k} & 0_{n_{x}\times n_{v}}\\ 0_{n_{v}\times n_{x}} & H \end{array}\right]. \end{array} \end{array}$$

where the weights satisfy ${\bar{\omega }}_{j}=1/\left(\pi^{n_{r}/2}\right)$, ${\bar{C}}_{j,k}$ consists of the first $mn_{r}$ columns of the numerical-derivative constraint matrix constructed for each measurement component $A_{j,k}^{a}$, and the column vectors of ${\bar{Q}}_{j,k}$ form an orthogonal basis for the null space of ${\bar{C}}_{j,k}$. As the basis vectors of the null space under the constraint matrix, ${\bar{Q}}_{k}$ can fully reveal the uncorrelated information that cannot be extracted directly. By identifying the basis directions of the uncorrelated information and performing projection mapping, the I-GCF achieves the extraction of all uncorrelated information under the imposed constraints.

**Figure 2:**
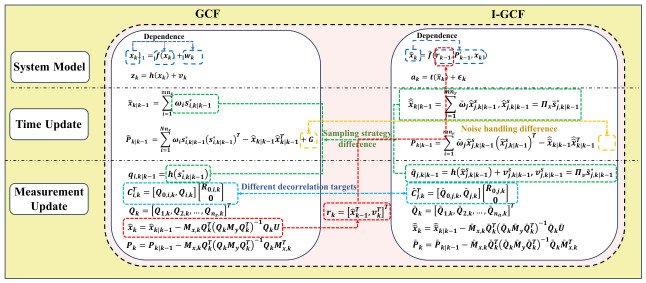
Comparison of the algorithmic procedures of GCF and I-GCF.

Figure 2 illustrates the fundamental differences between I-GCF and GCF in key algorithmic steps, including system modeling, time update, and measurement update. In the “System Model” part, the forward GCF performs recursion directly based on the physical state evolution, i.e., $x_{k+1}=f\left(x_{k}\right)+w_{k}$, whereas the I-GCF no longer directly depends on the physical dynamic system, but instead constructs an implicit inverse state transition relationship by characterizing the opponent’s forward filtering process, and explicitly introduces the augmented variable $r_{k}={\left[{\hat{x}}_{k-1},v_{k}\right]}^{T}$ for recursion. This part clearly reflects the fundamental difference in “Dependence” shown in the figure, namely, that GCF performs physical state estimation, whereas I-GCF operates at the cognitive estimation level. In the “Time Update” part, the forward GCF directly performs deterministic sampling and propagation in the original state space to obtain the predicted state and covariance. In contrast, the I-GCF conducts sampling in an augmented space, jointly propagates the state and noise, and extracts the state components through projection operations, corresponding to the difference indicated by “Sampling Strategy Difference”. In the “Measurement Update” part, although both methods extract decorrelated information via null-space projection, their observation domains are different: one originates from physical observations, whereas the other is derived from the mapping of the opponent’s estimated results. Moreover, for the GCF, the null-space basis is constructed from forward observation constraints, whereas for the I-GCF, it is derived from inverse observations together with augmented-space constraints, as explicitly indicated by “Different decorrelation targets”.

**Remark 4**  (Difference from I-EKF)**.** *The I-EKF handles nonlinear systems through Taylor-series expansion [3]. When the system is highly nonlinear, it not only causes the loss of higher-order nonlinear information but also tends to suffer from divergence. By contrast, the I-GCF employs deterministic sampling to address nonlinear scenarios. On the one hand, it avoids local linear approximation of the nonlinear system and reduces the risk of overall algorithmic divergence. On the other hand, such a moment-approximation method can effectively preserve higher-order moment information and reduce information loss.*

**Remark 5**  (Difference from I-UKF, I-CKF, and I-QKF)**.** *The I-UKF [7], I-CKF, I-QKF [13], and I-GCF are all inverse nonlinear filtering methods based on deterministic sampling and moment approximation. For the I-UKF, I-CKF, and I-QKF, the core of their nonlinear processing mechanism lies in generating a finite number of representative sample points via deterministic sampling, propagating them through nonlinear mappings, and approximating key statistical quantities, such as the posterior mean, covariance, and state-observation cross-covariance, through weighted summation, thereby enabling the handling of nonlinear information and mitigating truncation errors caused by local linearization. These three methods differ in their deterministic sampling rules, i.e., in the construction of representative sample points, and thus may exhibit different estimation performance under different system environments. However, the improvements of such methods are essentially limited to enhancing the extraction of low-order moment information by adjusting sampling strategies, and their information utilization framework does not go beyond moment approximation itself. In contrast, the I-GCF not only employs deterministic sampling as a tool for extracting low-dimensional moment information, but also treats it as a carrier for subsequent generalized conversion. After extracting low-order statistical information, it further introduces a generalized optimal conversion function, through which, together with null-space projection, it extracts uncorrelated information that is difficult to obtain solely via moment approximation, thereby enabling the exploration of higher-level nonlinear observation information. The non-restrictive nature of the deterministic sampling scheme in the I-GCF allows it to be compatible with the sampling strategies adopted by the I-UKF, I-CKF, and I-QKF, while also enabling the incorporation of other deterministic sampling schemes in different nonlinear scenarios. The fundamental advantage of the I-GCF over the I-UKF, I-CKF, and I-QKF does not lie in adopting a more advanced sampling rule, but in breaking through the limitation of conventional deterministic sampling methods that rely solely on low-order moment approximation, thereby achieving more sufficient extraction and utilization of nonlinear information. Furthermore, when the I-GCF adopts the sampling rules of the I-UKF, I-CKF, or I-QKF, it can also be regarded as a unified enhanced framework of these classical moment-based inverse filtering methods.*

**Remark 6**  (State update equation of I-GCF)**.** *The inverse state update Equation *(47)* and covariance update Equation *(48)* differ significantly in form from those of conventional inverse filters. This does not mean that the I-GCF modifies the update mechanism itself. Rather, after the generalized optimal conversion, most of the information in conventional inverse estimators, such as measurement correction, is represented by transformed parameters rather than by conventional state-form expressions. Nevertheless, the essential principle remains the same, since both follow the LMMSE estimation philosophy.*

**Remark 7**  (Comparison between GCF and I-GCF)**.** *Although the I-GCF is inspired by the forward GCF in algorithm derivation, due to the high complexity of the inverse inference task, the two methods exhibit fundamental differences in both theoretical interpretation and algorithmic mechanisms, as illustrated in Figure 2, and these differences can be summarized as follows:*

***(1)***  ***Estimation hierarchy and object:*** *In adversarial game scenarios, the forward GCF is usually deployed by the opponent to directly infer the defender’s objective physical motion state from sensor measurements, and thus belongs to direct state estimation in the physical domain. In contrast, the I-GCF is deployed by the defender. It is no longer governed directly by objective physical dynamics, but instead reconstructs the internal posterior output generated when the opponent executes forward filtering. This “estimation of an estimation result” constitutes a highly complex inference behavior in the cognitive domain.*
***(2)***  ***Dynamic system:*** *The state evolution of the forward GCF depends purely on the kinematic or dynamic equations of the objective physical environment, e.g., $x_{k+1}=f\left(x_{k}\right)+w_{k}$. By contrast, the dynamic system of the I-GCF is not derived entirely from physical facts; part of it is established on the algorithmic logic of the corresponding forward filter. Since the defender cannot access the opponent’s actual sensor observations, the I-GCF regards the “output generated by simulating one forward GCF update” as an abstract inverse state transition mapping.*
***(3)***  ***State dimension and augmented sampling treatment:*** *When the forward GCF employs deterministic sampling to extract nonlinear information, it operates directly in the original state space of dimension $n_{x}$. In the I-GCF, however, because the opponent’s random measurement noise $v_{k}$ exhibits a highly complex nonadditive effect during the simulated forward update, the I-GCF must construct an augmented state variable $r_{k}={\left[{\hat{x}}_{k-1}^{T},v_{k}^{T}\right]}^{T}$ that includes both the estimated state at the previous time step and the measurement noise. This forces the generalized optimal conversion and deterministic sampling of the I-GCF to be carried out in a higher-dimensional augmented space of dimension $n_{r}=n_{x}+n_{v}$.*
***(4)***  ***Noise treatment:*** *The noises encountered in the forward GCF can generally be handled as additive direct noises. In contrast, some of the noises involved in the I-GCF are complex nonadditive noises, which must be treated through augmented sampling and subsequent decomposition.*

## 5. Time Complexity Analysis

In practical adversarial environments, the time complexity of a filtering algorithm is also an important criterion for determining whether the algorithm should be adopted [22]. To this end, this section derives the time complexities of the forward GCF and the I-GCF. Considering the uncertainty in the actual computational cost caused by the non-fixed deterministic sampling scheme, the final time-complexity results are presented in the form of general expressions parameterized by the sampling complexity.

In the following analysis, $n_{x}$ denotes the state dimension, $n_{z}$ denotes the forward observation dimension, and $n_{a}$ denotes the inverse observation dimension. Let the function representing the number of sampling points generated by a deterministic sampling rule in a *d*-dimensional augmented space be denoted by

(50) $$N_{s}\left(d\right),$$

whose specific form varies with the adopted deterministic sampling scheme. For example, under the GHQ rule, $N_{s}\left(d\right)=m^{d}$, where *m* is the number of quadrature nodes in each dimension, and under the unscented transform, $N_{s}\left(d\right)=2d+1$. The time complexity of performing one deterministic sampling procedure in a *d*-dimensional space, including sampling-point generation, sample propagation, and weight accumulation, is denoted by

(51) $$C_{s}\left(d\right).$$

### 5.1. Time Complexity of GCF

The time complexity of the GCF mainly arises from two parts: deterministic sampling and constraint-matrix construction, together with matrix computations. For deterministic sampling, the sampling dimension is determined by the state dimension, i.e., $d_{f}=n_{x}$. The corresponding sampling complexity is

(52) $$C_{s}\left(n_{x}\right).$$

For the part of constraint-matrix construction and matrix computation, the major computational cost is related to the products of matrix dimensions, and the scale of these products is determined by the size of the constraint matrices. Since the scale of the constraint matrices is proportional to the number of sampling points, their dimension can be expressed as

(53) $$N_{s}\left(n_{x}\right)\times \left(N_{s}\left(n_{x}\right)+1\right),$$

and the complexity of this part can therefore be written as

(54) $$O\;\left(N_{s}\left(n_{x}\right)\left(N_{s}\left(n_{x}\right)+1\right)\right).$$

Since complexity analysis usually concerns only the dominant growth order as the problem size tends to infinity, while ignoring lower-order and constant terms [23], this complexity can be simplified as

(55) $$O\;\left(N_{s}\left(n_{x}\right)\left(N_{s}\left(n_{x}\right)+1\right)\right)≍O\;\left(N_{s}{\left(n_{x}\right)}^{2}\right).$$

Because the deterministic sampling part and the constraint-matrix construction part are independent of each other and do not affect each other’s computational complexity, the total time complexity of the GCF is

(56) $$T_{\mathrm{GCF}}=O\;\left(C_{s}\left(n_{x}\right)+N_{s}{\left(n_{x}\right)}^{2}\right).$$

### 5.2. Time Complexity of I-GCF

The algorithmic idea of the I-GCF is consistent with that of the GCF, and its computational complexity likewise consists of two parts: deterministic sampling and matrix construction together with matrix computations. For constraint-matrix construction, the computational complexity of the I-GCF is similar to that of the forward GCF, as both are determined by the scale of the constraint matrices. Since the inverse state dimension is $n_{r}$, the time complexity of this part can be calculated in the same way as in (55), and can be expressed as

(57) $$O\;\left(N_{s}{\left(n_{r}\right)}^{2}\right).$$

In the deterministic sampling part, the I-GCF performs deterministic sampling on the augmented variable, and the sampling dimension is therefore $d_{a}=n_{x}+n_{v}=n_{r}$. Accordingly, the number of sampling points is

(58) $$N_{s}\left(n_{r}\right).$$

Meanwhile, during each sampling process, every sampling point must undergo one forward simulation through the inverse state transition equation, which introduces one additional forward complexity term as in (56). Therefore, the time complexity of the complete deterministic sampling part can be expressed as

(59) $$O\left(N_{s}\left(n_{r}\right)\left(C_{s}\left(n_{x}\right)+N_{s}{\left(n_{x}\right)}^{2}\right)\right).$$

Since the two computational parts of the I-GCF are also independent of each other, the overall time complexity of the I-GCF can be written as

(60) $$T_{IGCF}=O\left(N_{s}\left(n_{r}\right)\left(C_{s}\left(n_{x}\right)+N_{s}{\left(n_{x}\right)}^{2}\right)+N_{s}{\left(n_{r}\right)}^{2}\right).$$

**Remark 8**  (Comparison between the time complexities of GCF and I-GCF)**.** *The sources of the time complexities of the GCF and the I-GCF are similar, since both arise from deterministic sampling and constraint-matrix construction together with matrix operations. However, in the deterministic sampling stage of the I-GCF, each sampling point requires one simulated forward GCF execution, resulting in an approximately multiplicative increase in time complexity. Consequently, the time complexity of the I-GCF is significantly higher than that of the GCF.*

**Remark 9**  (Time complexity under GHQ sampling)**.** *The deterministic sampling scheme used in the algorithm derivation and numerical simulations in this paper is GHQ sampling. Accordingly, this paper further computes the explicit time complexities of the GCF and the I-GCF under this sampling scheme. When GHQ sampling is adopted, $N_{s}\left(d\right)=m^{d}$, and thus*

*(1)*  *GCF:*
  $$T_{GCF}=O\left(m^{n_{x}}+m^{2n_{x}}\right).$$
*(2)*  *I-GCF:*
  $$T_{IGCF}=O\left(m^{n_{r}}\left(m^{n_{x}}+m^{2n_{x}}\right)+m^{2n_{r}}\right).$$

## 6. Numerical Experiments

Current inverse nonlinear filtering methods for handling system nonlinearity can mainly be categorized into two mainstream technical approaches: local linearization based on Taylor expansion, and moment approximation based on deterministic sampling [7]. The former is represented by the I-EKF, which performs local linearization of nonlinear systems via first-order Taylor expansion, and is one of the most classical and widely used methods in inverse nonlinear filtering. The latter is represented by the I-UKF and I-CKF, which employ different deterministic sampling mechanisms and can comprehensively characterize the capability of mainstream inverse filtering methods within the moment approximation framework in exploiting nonlinear information. Therefore, the I-EKF, the I-UKF, and I-CKF [13] can be regarded as representative methods of the two mainstream technical routes, namely, local linearization and deterministic sampling-based moment approximation, respectively.

This paper verifies the effectiveness of the I-GCF by comparing its estimation errors with those of the I-CKF, I-UKF, and I-EKF under both normal operating conditions and model-mismatch scenarios. The number of Monte Carlo runs for all systems is set to 200, and the I-GCF adopts the GHQ rule [24] for deterministic sampling.

### 6.1. Chaotic Adversarial System with Nonlinear Range Sensors

The discrete Lorenz system is often used to simulate turbulent characteristics in complex meteorological sensor networks and adversarial aircraft trajectories with strong unpredictability. Here, a discretized three-dimensional Lorenz chaotic system is adopted as the kinematic model of the defender for numerical simulation [25]. All initialization parameters in the system follow the standard settings in [13]. The parameters of the UKF and I-UKF are also set according to this reference as $\kappa =\bar{\kappa }=-1$, while the CKF and I-CKF do not require additional tuning parameters. For the I-GCF, the one-dimensional number of sampling nodes *N* in the GHQ sampling scheme is set to 3.

The state transition equation is given by

(61) $$x_{k}=f\left(x_{k-1}\right)+Mw_{k-1},$$

where the state transition function $f(x_{k-1})$ is

(62) $$f\left(x_{k-1}\right)=\left[\begin{array}{c} x_{k-1}^{\left(1\right)}+\Delta t\;r_{1}\left(-x_{k-1}^{\left(1\right)}+x_{k-1}^{\left(2\right)}\right)\\ x_{k-1}^{\left(2\right)}+\Delta t\left(r_{2}x_{k-1}^{\left(1\right)}-x_{k-1}^{\left(2\right)}-x_{k-1}^{\left(1\right)}x_{k-1}^{\left(3\right)}\right)\\ x_{k-1}^{\left(3\right)}+\Delta t\left(-r_{3}x_{k-1}^{\left(3\right)}+x_{k-1}^{\left(1\right)}x_{k-1}^{\left(2\right)}\right) \end{array}\right].$$

Here, $x_{k}\in R^{3}$, and the process noise satisfies $w_{k}∼N\left(0,G\right)$, with

(63) $$M=\left[\begin{array}{c} 0\\ 0\\ 0.5 \end{array}\right],\;G=\Delta t.$$

The system parameters are set as Δt=0.01, $r_{1}=10$, $r_{2}=28$, and $r_{3}=8/3$.

The opponent usually deploys sensors such as ultra-wideband (UWB) or multistatic radar networks to observe the defender. The resulting sensor observation equation is a typical nonlinear range measurement model with spatial offset, given by

(64) $$z_{k}=h\left(x_{k}\right)+0.065\;v_{k},$$

where the observation function $h(x_{k})$ is

(65) $$h\left(x_{k}\right)=\Delta t\sqrt{{\left(x_{k}^{\left(1\right)}-0.5\right)}^{2}+{\left(x_{k}^{\left(2\right)}\right)}^{2}+{\left(x_{k}^{\left(3\right)}\right)}^{2}},$$

and the measurement noise satisfies $v_{k}∼N\left(0,H\right)$, with H=Δt.

Based on observations from its own deployed sensors, the defender constructs the following inverse observation model for the opponent’s forward filtering estimate ${\hat{x}}_{k}$:

(66) $$a_{k}=t\left({\hat{x}}_{k}\right)+0.1\;\epsilon_{k},$$

where the defender’s observation function $t({\hat{x}}_{k})$ is

(67) $$t\left({\hat{x}}_{k}\right)=\Delta t\sqrt{{\left({\hat{x}}_{k}^{\left(1\right)}\right)}^{2}+{\left({\hat{x}}_{k}^{\left(2\right)}-0.5\right)}^{2}+{\left({\hat{x}}_{k}^{\left(3\right)}\right)}^{2}},$$

and $\epsilon_{k}∼N\left(0,\Sigma_{\epsilon }\right)$, with $\Sigma_{\epsilon }=\Delta t$.

The initial system state is set as

(68) $$x_{0}=\left[\begin{array}{c} -0.2\\ -0.3\\ -0.5 \end{array}\right],\;P_{0}={\bar{P}}_{0}=0.35\;I.$$

The initial estimate of the opponent’s forward filter is

(69) $${\hat{x}}_{0}=\left[\begin{array}{c} 1.35\\ -3.6\\ 2.8 \end{array}\right].$$

Given the augmented state dimension $n_{r}=4$, the state dimension $n_{x}=3$, and the number of GHQ sampling nodes equal to 3, the time complexity of the I-GCF for this system can be obtained from (60) as

$$T_{IGCF}=O\left(3^{4}\left(3^{3}+3^{6}\right)+3^{8}\right)\approx O\left(3^{10}\right).$$

For this system, the performances of the I-GCF, I-UKF, I-CKF, and I-EKF are compared.

**Figure 3:**
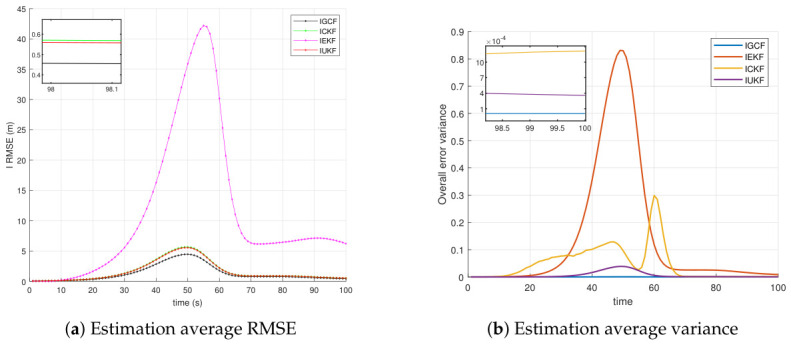
Estimation Performance under matched forward-filter assumptions in the Lorenz system.

For this type of nonlinear spatial range-sensor system, under the condition that the forward assumptions of all inverse filters are satisfied, Figure 3 presents the variations of the average root-mean-square error (RMSE) (a) and the average estimation variance (b) of the I-GCF, I-CKF, I-EKF, and I-UKF in the three-dimensional Lorenz chaotic system under Matlab (2022b) with 200 Monte Carlo simulations. A lower average RMSE and a smoother overall curve indicate smaller estimation error, higher estimation stability, and better overall performance. A lower average estimation variance and a smoother curve indicate smaller estimation uncertainty and weaker fluctuation, thereby reflecting higher stability and overall performance. From Figure 3a, it can be observed that, within the main error growth interval (approximately 40~60 s), the RMSE of the I-GCF remains consistently lower than those of the I-UKF, I-CKF, and I-EKF. At the main peak (around t≈50s), the I-GCF achieves a reduction of approximately 20% compared with the I-UKF and I-CKF, and nearly one order of magnitude compared with the I-EKF. From the perspective of fluctuation characteristics, the I-GCF, I-UKF, and I-CKF rapidly converge to a low-error region after 60s, while the I-GCF consistently maintains the lowest error with a stable trend, whereas the I-EKF remains at a relatively high level after the peak and exhibits significantly larger fluctuations. The zoomed-in results further show that, near the final stage, the steady-state error of the I-GCF is still lower than those of the I-UKF and I-CKF. This indicates that the I-GCF effectively suppresses error propagation by extracting and utilizing higher-dimensional information from observations, thereby improving estimation accuracy and overcoming some limitations of conventional inverse filters. From Figure 3b, it can be observed that the I-GCF maintains the lowest variance level throughout the entire time interval, with its curve almost aligned with the horizontal axis, and still significantly lower than those of the I-EKF, I-CKF, and I-UKF in the zoomed-in view. This indicates that the I-GCF not only achieves a smaller average estimation error, but also exhibits a more concentrated error distribution and weaker stochastic fluctuations, thereby demonstrating stronger estimation stability. In contrast, the I-EKF shows the most significant increase in variance in the middle stage, reaching the highest peak around t≈50, indicating its high sensitivity to strongly nonlinear phases. The I-CKF exhibits lower overall fluctuation than the I-EKF, but still shows noticeable local peaks in the middle and later stages, particularly around t≈60. The I-UKF performs better than the I-EKF and I-CKF, with a generally lower variance curve and only slight increases near the main peak; however, its steady-state variance remains higher than that of the I-GCF, indicating that its error dispersion is not minimized. These results demonstrate that the I-GCF maintains the smallest error variance and the most stable evolution trend in strongly nonlinear, strongly coupled, and noise-sensitive systems such as the Lorenz system, thereby exhibiting superior statistical stability and robustness.

**Figure 4:**
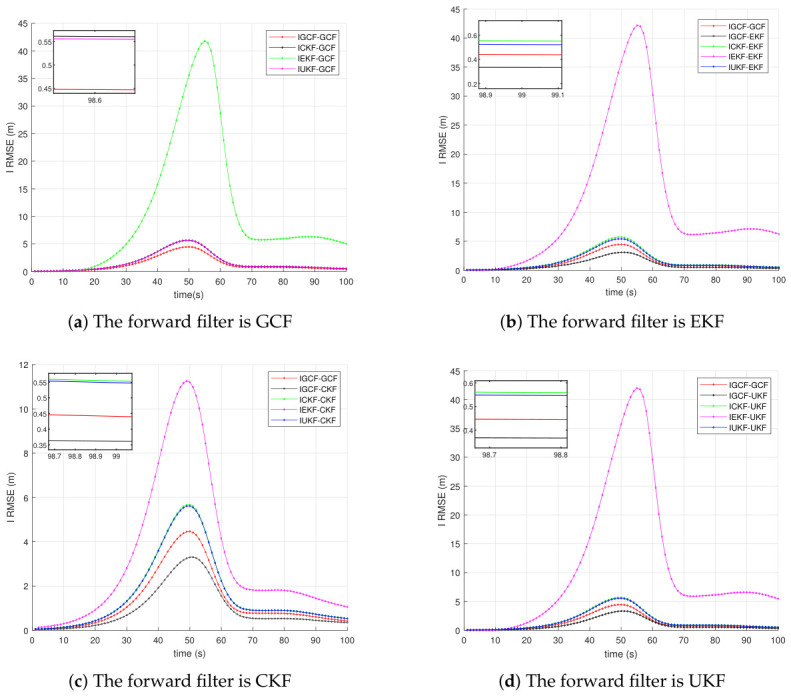
Estimation RMSE under different conditions in the Lorenz system.

Figure 4 presents the average RMSE results of inverse filters in the Lorenz chaotic system, obtained under Matlab (2022b) with 200 Monte Carlo simulations, where the actual forward filters are GCF, EKF, CKF, and UKF, respectively. The labels in the figure indicate the adopted inverse filter and its corresponding actual forward filter. For example, ICKF-GCF denotes that when the actual forward filter is GCF, the I-CKF is used for inverse estimation. It can be observed that, regardless of whether the forward filter satisfies the forward-matching assumption of the inverse filter, the I-GCF consistently achieves the lowest estimation error and also exhibits the lowest error peak. In particular, in Figure 4c, around t≈50s, the main peak of IGCF-CKF is approximately 3.2∼3.4 m, while the peaks of ICKF-CKF and IUKF-CKF are both approximately 5.5∼5.7 m, and the peak of IEKF-CKF further increases to about 11m. This implies that, although ICKF-CKF satisfies the forward-filter matching assumption, the peak error of IGCF-CKF is still reduced by approximately 35∼40%, and by more than 70% compared with IEKF-CKF. These results indicate that the advantage of the I-GCF does not rely on strict model matching, but rather stems from its more effective utilization of decorrelated information in inverse observations. Even when other inverse filters satisfy the forward-matching assumption, the I-GCF still achieves lower average error and smaller peak error, thereby demonstrating stronger overall inverse estimation capability.

**Figure 5:**
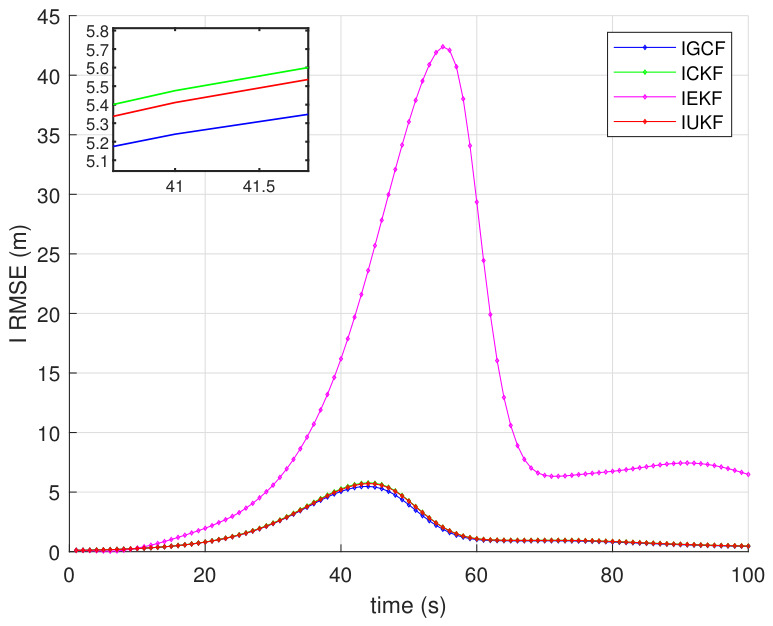
Estimation RMSE in the Lorenz system under initial-parameter mismatch conditions.

To further validate the estimation capability of the I-GCF under practical mismatch conditions, this paper considers scenarios in which the defender has erroneous prior knowledge of the initial state and initial covariance, and conducts inverse filtering estimation and performance comparison. Figure 5 presents the RMSE variations of I-GCF, I-CKF, I-EKF, and I-UKF in the three-dimensional Lorenz chaotic system under incorrect initial state and covariance conditions, obtained under Matlab (2022b) with 200 Monte Carlo simulations. The erroneous initial state and initial covariance are randomly generated from uniform distributions. It can be observed that the overall iterative trends of all filters remain consistent with those under correctly specified parameters, while the I-GCF still maintains the lowest estimation error and the smoothest error variation. These results indicate that the I-GCF possesses strong error correction capability and can effectively adapt to practical environments.

**Figure 6:**
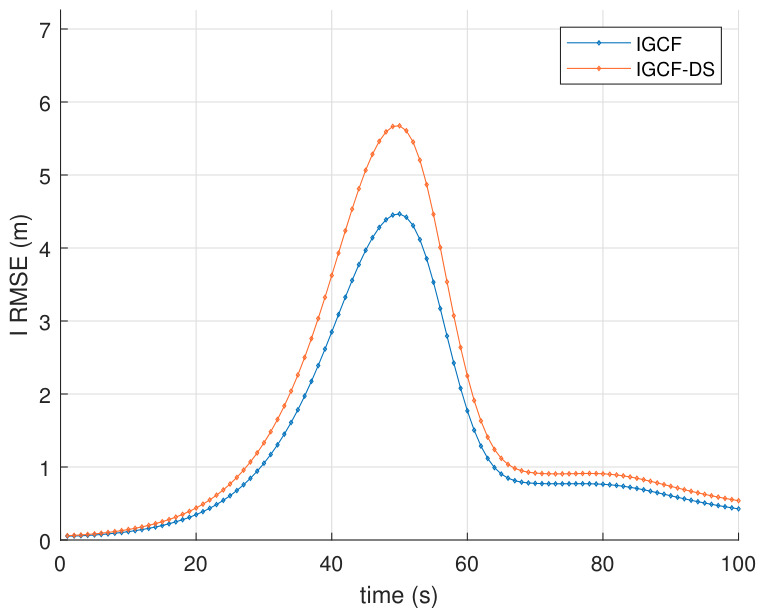
Ablation study of I-GCF in the Lorenz system.

To further validate the performance improvement brought by the generalized decorrelation-based transformation to the I-GCF, ablation experiments are conducted in this paper to compare the estimation error between the full I-GCF and the I-GCF without the generalized decorrelation component, as shown in Figure 6. The figure presents the average RMSE results of the I-GCF and the I-GCF without the generalized decorrelation component in the Lorenz chaotic system, obtained under Matlab (2022b) with 200 Monte Carlo simulations. Here, “IGCF-DS” denotes the I-GCF without the generalized decorrelation component. From the figure, the overall error evolution trends of the two methods are consistent, both exhibiting significant error peaks in the middle stage and gradually converging in the later stage; however, the I-GCF maintains a consistently lower RMSE level throughout the entire time interval. In particular, near the error peak, the maximum RMSE of the I-GCF is approximately 4.47 m, whereas that of the I-GCF-DS is about 5.67 m, indicating a reduction of approximately 21% for the former. This demonstrates that, in the most nonlinear and most challenging estimation stage of the system, the I-GCF can more effectively suppress error amplification. From the experimental observations, the consistently superior performance of the I-GCF over the I-GCF-DS indicates that the additional mechanism in the complete algorithm not only enhances the estimation capability in highly nonlinear regions, but also improves the overall error convergence quality. It can therefore be concluded that, for such systems, the generalized decorrelation-based transformation plays a significant role in improving estimation performance.

**Figure 7:**
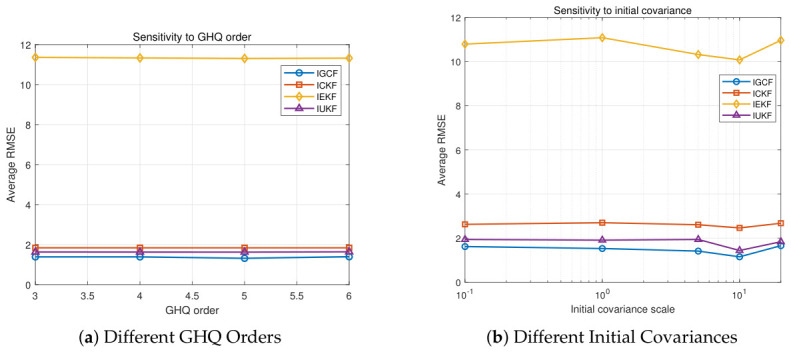
Sensitivity of I-GCF Reflected by Average RMSE in the Lorenz system.

To further analyze the parameter sensitivity of the I-GCF in the strongly nonlinear Lorenz system, experiments are conducted in this paper from two key factors in filtering estimation, namely, deterministic sampling accuracy and initialization conditions, as shown in Figure 7. The figure presents the estimation error variations of I-GCF, I-CKF, I-EKF, and I-UKF in the three-dimensional Lorenz chaotic system under different GHQ sampling orders (a) and different initial covariance settings (b), obtained under Matlab (2022b). For each parameter configuration, 200 Monte Carlo simulations are performed, and the average RMSE shown on the vertical axis is the overall mean of all simulation results for the setting. In Figure 7a, the errors of all methods remain relatively stable as the GHQ order varies; however, the I-GCF consistently achieves the lowest RMSE level and exhibits low sensitivity to changes in the sampling order. Compared with I-CKF and I-UKF, the average error of I-GCF is reduced by approximately 10–20%, while compared with I-EKF, the reduction exceeds 85%. In Figure 7b, all methods exhibit different levels of sensitivity to initialization uncertainty. As the scale of the initial covariance varies, the RMSE of the I-GCF remains consistently low, with relatively small fluctuations. In contrast, the errors of I-CKF and I-UKF vary more significantly with the covariance changes, whereas I-EKF consistently maintains a higher error level with more pronounced fluctuations. These results indicate that the I-GCF can effectively suppress the impact of initialization errors on the subsequent estimation process. The two sets of experiments jointly demonstrate, from the perspectives of sampling parameters and initialization conditions, that the I-GCF exhibits low sensitivity to key parameter perturbations and achieves higher stability in strongly nonlinear dynamic systems. This advantage theoretically stems from its generalized optimal conversion mechanism, which enables more effective utilization of higher-order nonlinear information in observations, thereby mitigating the impact of parameter variations on estimation accuracy.

**Figure 8:**
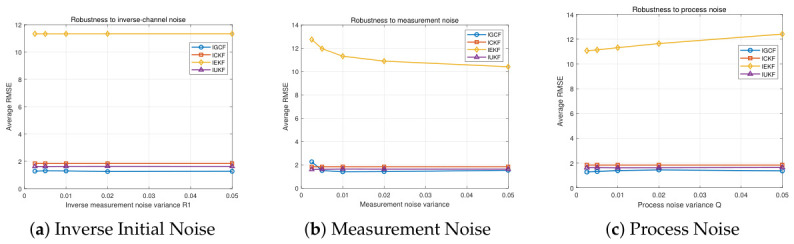
Average RMSE under Different Noise Conditions in the Lorenz System.

To evaluate the noise robustness of the I-GCF in the strongly nonlinear Lorenz system, parameter perturbation experiments are conducted from three aspects, namely, inverse initial noise, measurement noise, and process noise, as shown in Figure 8. The figure presents the estimation error variations of I-GCF, I-CKF, I-EKF, and I-UKF in the Lorenz system under different inverse initial noise (a), measurement noise (b), and process noise (c), obtained under Matlab (2022b). For each noise configuration, 200 Monte Carlo simulations are performed, and the average RMSE shown on the vertical axis is the overall mean of the simulation results for that setting. In Figure 8a, the error variations of all methods are relatively smooth, indicating that the variation of the inverse initial covariance has a limited impact on all filters in this system. In Figure 8b, as the noise variance increases from approximately 0.0025 to 0.05, the RMSE of the I-GCF decreases from about 1.84 to 1.53, showing an overall gradual decreasing trend; in contrast, the RMSE of I-CKF and I-UKF remains relatively stable within the range of 1.63–1.84 with only slight fluctuations, while the I-EKF, although exhibiting a slight decrease with increasing noise, consistently maintains a relatively high error level within 10.4–12.7. It can be observed that the I-GCF exhibits a certain degree of sensitivity to measurement noise in the low-noise region; however, as the noise level increases, the RMSE of the I-GCF still consistently remains at the lowest level. In Figure 8c, the process noise has a significant impact on the I-EKF, whereas the I-GCF, I-UKF, and I-CKF remain relatively stable, and the RMSE of the I-GCF consistently maintains the lowest level among all methods. Overall, the three sets of experimental results consistently demonstrate that, under different types of noise perturbations, the I-GCF can maintain low and stable estimation errors. This indicates that the I-GCF exhibits strong noise robustness in the strongly nonlinear Lorenz system, and its advantage theoretically stems from the generalized optimal conversion mechanism, which effectively mitigates the impact of noise on the nonlinear estimation process, thereby maintaining stable performance under different noise conditions.

Table 2 presents the average execution time of different filters in the three-dimensional Lorenz chaotic system under matched conditions. The results show that all filtering methods can operate normally under matched conditions. Among them, the I-EKF and I-UKF exhibit faster computational speed, while the execution time of the I-GCF and I-CKF is relatively higher.

### 6.2. FM Signal Interception and Phase Estimation Adversarial System Based on SDR Sensors

Frequency modulation (FM) signal demodulation is a classical nonlinear signal-processing problem in software-defined radio (SDR), passive radar, and acoustic sensor networks. Consider the discrete-time nonlinear system model of an FM demodulator [26]. All initialization parameters in the system follow the standard settings in [13]. The parameters of the UKF and I-UKF are also set according to this reference as $\kappa =\bar{\kappa }=-1$, while the CKF and I-CKF do not require additional tuning parameters. For the I-GCF, the one-dimensional number of sampling nodes *N* in the GHQ sampling scheme is set to 4. The system state transition equation is given by

(70) $$x_{k}=Fx_{k-1}+Mw_{k-1},$$

where the process noise satisfies $w_{k}∼N\left(0,G\right)$, with G=0.01.

The system state is defined as

(71) $$x_{k}≜\left[\begin{array}{c} \lambda_{k}\\ \theta_{k} \end{array}\right],$$

where $\lambda_{k}$ represents the instantaneous frequency offset and $\theta_{k}$ represents the instantaneous phase in the polar coordinate system. During the simulation process, periodic normalization is applied to $\theta_{k}$ so that it always remains within the interval [−π,π], thereby avoiding model mismatch caused by phase accumulation.

The state transition matrix is

(72) $$F=\left[\begin{array}{cc} \exp\;\left(-\frac{T}{\beta }\right) & 0\\ -\beta \exp\;\left(-\frac{T}{\beta }\right)-1 & 1 \end{array}\right],$$

where the sampling interval is $T=\frac{2\pi }{16}$ and the inertial time constant is β=100. The parameter β is used to characterize the inertial property of the system, whereas *T* determines the discretization accuracy. Their coupled effect determines the evolution characteristics of the frequency offset $\lambda_{k}$ and the phase $\theta_{k}$, thereby affecting the dynamic behavior of the system state.

The process-noise input matrix is

(73) $$M=\left[\begin{array}{c} 1\\ -\beta \end{array}\right],$$

which describes how the random noise $w_{k}$ affects the system state $x_{k}$, thereby realizing the mapping from noise to the state space.

The opponent sensor performs frequency conversion on the received signal, and its observation equation is defined as

(74) $$z_{k}=\sqrt{2}\left[\begin{array}{c} \sin\theta_{k}\\ \cos\theta_{k} \end{array}\right]+v_{k},$$

where the observation noise satisfies

$$v_{k}∼N\;\left(0_{2\times 1},H\right),$$

with

$$H=\left[\begin{array}{cc} 1 & 0\\ 0 & 1 \end{array}\right].$$

The observation $z_{k}$ represents the phase measurement data in the Cartesian coordinate system acquired by the sensor through observation of the phase angle $\theta_{k}$, and is the information directly available to the opponent. In practical implementation, the angle $\theta_{k}$ also needs to be normalized so that it always lies within the interval [−π,π].

The defender’s observation model is defined as

(75) $$a_{k}={\hat{\lambda }}_{k}^{2}+\epsilon_{k},$$

where

$$\epsilon_{k}∼N\left(0,\Sigma_{\epsilon }\right),\;\Sigma_{\epsilon }=5,$$

and ${\hat{\lambda }}_{k}$ denotes the estimated component of the opponent’s state estimate ${\hat{x}}_{k}$.

The initial system state is set as

(76) $$x_{0}=\left[\begin{array}{c} \lambda_{0}\\ \theta_{0} \end{array}\right],$$

where $\lambda_{0}∼N\left(0,1\right)$ and $\theta_{0}∼U\left(-\pi ,\pi \right)$. Randomizing the initial conditions helps avoid the influence of individual events on the experimental results, thereby enabling a more comprehensive evaluation of the overall performance of the filtering algorithms.

The initial covariance of the opponent’s estimate is set as

(77) $$P_{0}=\left[\begin{array}{cc} 1 & 0\\ 0 & 4 \end{array}\right],$$

and the initial covariance of the defender is

(78) $${\bar{P}}_{0}=\left[\begin{array}{cc} 1 & 0\\ 0 & 4 \end{array}\right].$$

To avoid the influence of rounding errors on the positive definiteness of the covariance matrix during numerical computation, a positive-definite perturbation term of $10^{-10}$ is added to the covariance matrix.

Given the augmented state dimension $n_{r}=4$, the state dimension $n_{x}=2$, and the number of GHQ sampling nodes equal to 4, the time complexity of the I-GCF for this system can be obtained from (60) as

$$T_{IGCF}=O\left(4^{4}\left(4^{2}+4^{4}\right)+4^{8}\right)\approx O\left(4^{8}\right).$$

**Figure 9:**
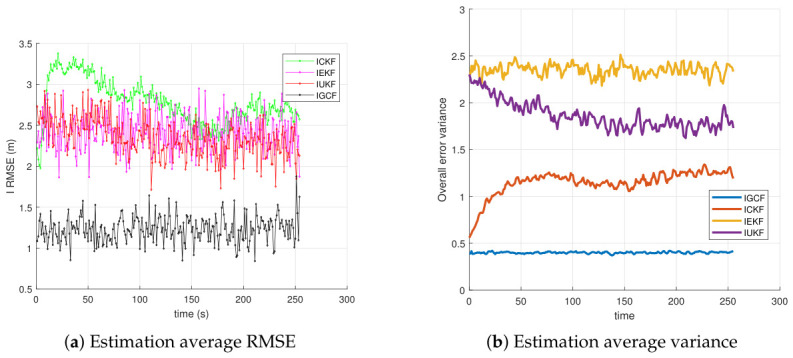
Estimation Performance under matched forward-filter assumptions in the FM demodulation oscillation system.

Figure 9 presents the average RMSE (a) and average estimation variance (b) of different inverse filters in the FM demodulation system under the matched forward-filter assumption, obtained under Matlab (2022b) with 200 Monte Carlo simulations. Similar to Figure 3, a lower average RMSE and a smoother curve indicate better inverse estimation performance. A lower average variance and a more stable curve also indicate better inverse estimation performance. From Figure 9a, the average RMSE of all inverse filters exhibits time-varying oscillatory characteristics, while significant differences can be observed in both error levels and fluctuation amplitudes among different methods. The I-GCF mainly oscillates within the range of approximately 1∼1.5 m, with the smallest fluctuation amplitude, indicating that it achieves the lowest overall average RMSE and maintains relatively smooth error variations over long-term simulations. In contrast, the error levels of the I-UKF and I-EKF are relatively close, mainly oscillating within the range of approximately 2∼3 m, with more pronounced fluctuations, indicating that these methods are more sensitive to system noise disturbances and nonlinear observation errors. Although the I-CKF shows relatively reduced oscillation in certain local intervals, its overall error band remains significantly higher than that of the I-GCF, and its fluctuation amplitude across the entire time domain is still large, resulting in inferior overall performance compared with the I-GCF. Although the state dimension of the FM demodulation system is lower than that of the Lorenz system, its observation model includes trigonometric phase functions and squared inverse observation relationships, and therefore still exhibits strong nonlinearity. Therefore, the advantage of the I-GCF in extracting more observation information through generalized optimal conversion remains highly significant. This figure demonstrates that the I-GCF is not only applicable to high-dimensional chaotic systems, but also achieves superior inverse estimation performance in low- and medium-dimensional systems with strongly nonlinear observations. From Figure 9b, it can be observed that the I-GCF maintains the lowest error variance throughout the entire time interval, with its curve remaining around approximately 0.4 and exhibiting only very small fluctuations, demonstrating the strongest stability and consistency. In contrast, the error variance of the I-CKF rapidly increases from approximately 0.55 in the initial stage and remains within the range of approximately 1.1–1.3 for most of the subsequent time, which is significantly higher than that of the I-GCF, indicating that although it can achieve effective inverse estimation under matched conditions, its sample dispersion remains relatively large and long-term stability is insufficient. The error variance of the I-UKF is generally within the range of approximately 1.7–2.3, showing some decrease in the early stage but remaining at a relatively high level afterward, indicating that although its estimation results exhibit certain consistency, the overall fluctuation remains significant. The I-EKF exhibits the highest error variance, remaining around approximately 2.3–2.5 over the entire time interval and significantly higher than the other three methods, indicating that local linearization methods are most prone to error diffusion and sample dispersion in nonlinear environments such as the FM system with trigonometric observation functions and squared inverse observation relationships. It is worth noting that, unlike the peak-type variance explosion observed in the Lorenz system, the variance curves in the FM system exhibit a long-term plateau-type difference, meaning that the differences among methods are not mainly reflected in instantaneous peaks but in the sustained level of statistical dispersion throughout the entire simulation period. In this sense, the advantage of the I-GCF becomes more evident, as it not only performs better at certain local time instants but also consistently maintains the lowest and most stable error variance over the entire time interval, indicating stronger overall suppression capability against observation noise, phase-coupled nonlinearity, and error propagation.

**Figure 10:**
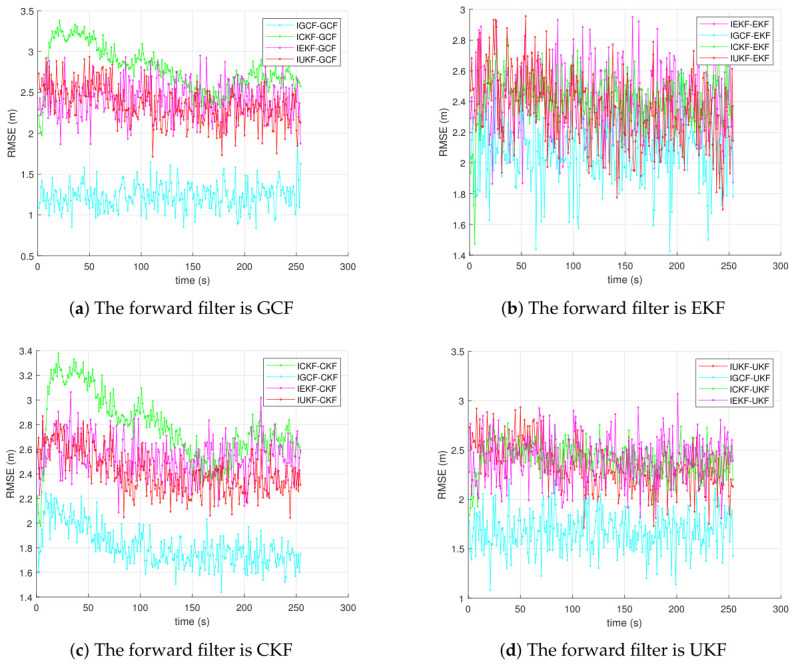
Estimation RMSE under different mismatch conditions in the FM demodulation oscillation system.

Figure 10 presents the average RMSE results of inverse filters in the FM demodulation oscillation system under different forward-filter mismatch conditions, obtained under Matlab (2022b) with 200 Monte Carlo simulations. In Figure 10a–d, the actual forward filters are GCF, EKF, CKF, and UKF, respectively. Figure 10a shows that, under the matched condition of the I-GCF model, the RMSE level of the I-GCF is significantly lower than those of the I-CKF, I-EKF, and I-UKF, indicating that it achieves the best baseline performance. Figure 10b shows that, when the actual forward filter is EKF, although the oscillation range of IGCF-EKF increases, it still maintains the lowest or near-lowest error level, and its overall performance is superior to IEKF-EKF. This indicates that, even under mismatch conditions, the I-GCF can compensate for the performance degradation caused by model inconsistency through its stronger information extraction capability. Figure 10c shows that, when the actual forward filter is CKF, although IGCF-CKF exhibits noticeable local fluctuation peaks, its overall error curve is still significantly lower than that of ICKF-CKF. Moreover, it gradually converges to a relatively stable oscillation range in the later stage, approximately within 1.5∼2 m, indicating that the I-GCF still possesses strong error suppression capability and steady-state robustness under CKF mismatch conditions. Similarly, in Figure 10d, when the actual forward filter is UKF, the average RMSE of IGCF-UKF is also lower than that of IUKF-UKF and other inverse filtering methods, further demonstrating that the I-GCF has good adaptability to different forward filter forms. From Figure 10, it can be concluded that, in the FM demodulation system with significant measurement nonlinearity and phase-coupling characteristics, the I-GCF maintains strong consistency and adaptability under various forward-filter mismatch conditions. In other words, even when the defender does not fully know the actual forward filter adopted by the opponent, the I-GCF can still extract more sufficient inverse estimation information from limited observations based on the generalized optimal conversion framework, thereby achieving lower average RMSE.

**Figure 11:**
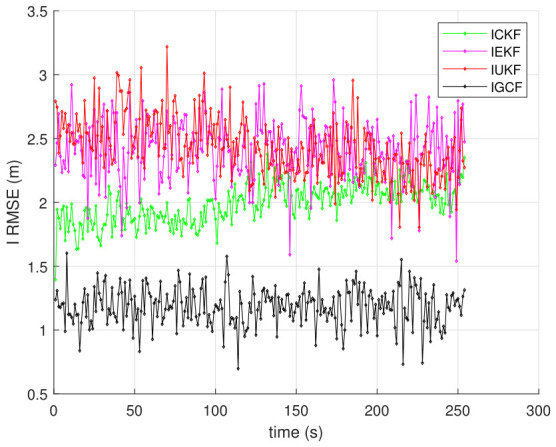
Estimation RMSE in the FM demodulation oscillation system under initial-parameter mismatch conditions.

Figure 11 presents the RMSE variations of I-GCF, I-CKF, I-EKF, and I-UKF in the FM demodulation oscillation system under incorrect initial state and covariance conditions, obtained under Matlab (2022b) with 200 Monte Carlo simulations. The erroneous initial state and initial covariance are randomly generated from uniform distributions. The I-GCF maintains its advantage, consistently achieving the lowest estimation error and the smallest error variation range. It is noteworthy that the I-CKF exhibits better estimation performance in this scenario compared with the model mismatch case, indicating that its performance is more susceptible to the influence of initial conditions and external estimation, and lacks the stability of the I-GCF.

**Figure 12:**
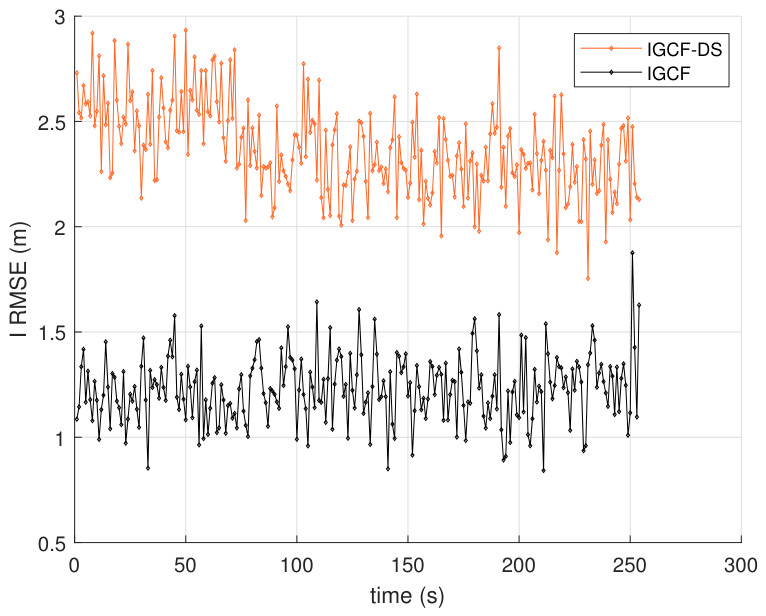
Ablation study of I-GCF in the FM demodulation oscillation system.

Figure 12 presents the average RMSE results of the I-GCF and the I-GCF without the generalized decorrelation component in the FM demodulation oscillation system, obtained under Matlab (2022b) with 200 Monte Carlo simulations. It can be observed that the IGCF-DS exhibits significantly larger oscillation ranges, variation amplitudes, and RMSE values compared with the I-GCF. This indicates that, in the ablation experiments of this system, the generalized decorrelation-based transformation also plays an important role in improving the performance of the I-GCF, which is consistent with the conclusions drawn from the Lorenz system. This further verifies the performance superiority brought by the generalized decorrelation-based transformation to the I-GCF.

**Figure 13:**
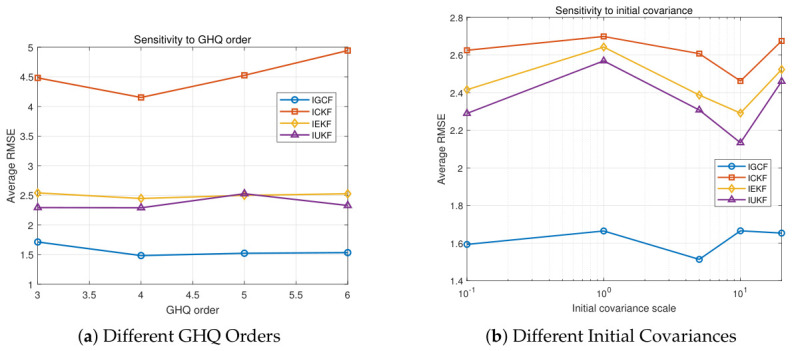
Sensitivity of I-GCF Reflected by Average RMSE in the FM demodulation oscillation system.

Figure 13 presents the estimation error variations of I-GCF, I-CKF, I-EKF, and I-UKF in the FM demodulation system under different GHQ sampling orders (a) and different initial covariance settings (b), obtained under Matlab (2022b). For each parameter configuration, 200 Monte Carlo simulations are performed, and the average RMSE shown on the vertical axis represents the overall mean of all simulation results under the corresponding setting. In Figure 13a, as the GHQ order increases, all methods exhibit a trend of slight improvement followed by stabilization; however, the I-GCF consistently maintains the lowest error level. Specifically, compared with I-CKF and I-UKF, the I-GCF achieves an average RMSE reduction of approximately 30–40% under different sampling orders; compared with I-EKF, the reduction is more significant, exceeding approximately 60%. In addition, the I-GCF exhibits stronger stability with respect to changes in the sampling order, and its error fluctuation amplitude is significantly smaller than that of other methods, being within approximately one-half of theirs. In Figure 13b, as the initial covariance increases, the errors of all methods exhibit a non-monotonic trend of first increasing, then decreasing, and finally stabilizing, reflecting the significant impact of initialization uncertainty on the inverse estimation process. Throughout the entire variation range, the I-GCF consistently maintains the lowest or near-lowest error level; compared with I-CKF and I-UKF, it achieves an average RMSE reduction of approximately 10–20%, while compared with I-EKF, the reduction is approximately 35–45%. More importantly, the I-GCF exhibits the smallest performance fluctuation under different initial covariance scales, with its error variation range significantly reduced, with the overall fluctuation amplitude being reduced by more than approximately 30% compared with other methods. In particular, even under large covariance conditions, such as 10 and above, the I-GCF is still able to maintain stable estimation performance. This indicates that conventional methods are prone to performance degradation under large initialization uncertainty due to linearization errors or sampling distribution dispersion, whereas the I-GCF effectively mitigates the amplification effect of initialization errors on posterior estimation. In such low- to medium-dimensional systems with strong nonlinearity, the I-GCF exhibits solid stability under perturbations of the two key factors in the filtering process and demonstrates low sensitivity to variations in these factors.

**Figure 14:**
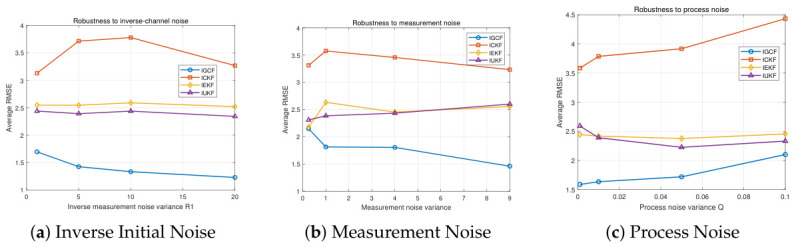
Average RMSE under Different Noise Conditions in the FM demodulation oscillation system.

Figure 14 presents the estimation error variations of I-GCF, I-CKF, I-EKF, and I-UKF in the FM demodulation system under different inverse initial noise (a), measurement noise (b), and process noise (c), obtained under Matlab (2022b). For each noise configuration, 200 Monte Carlo simulations are performed, and the average RMSE shown on the vertical axis represents the overall mean of all simulation results under the corresponding setting. From the overall trend, as the noise level varies, the estimation errors of all methods exhibit different degrees of fluctuation; however, the I-GCF consistently maintains the lowest or near-lowest RMSE level. In Figure 14a, the I-GCF achieves an error reduction of approximately 50–60% compared with I-CKF, and approximately 30–45% compared with I-UKF and I-EKF. Moreover, its error varies monotonically and gradually with noise, demonstrating strong stability. In Fmonotonically and gradually with noiseig. Figure 14b, the RMSE variation of the I-GCF is significantly smaller than that of other methods, with the overall fluctuation reduced by more than approximately 30%, while still maintaining an accuracy advantage of approximately 20–40%, indicating stronger adaptability to observation uncertainty. In Figure 14c, although the errors of all methods increase with the noise level, the I-GCF exhibits the smallest growth rate, and its average error is still reduced by approximately 25–50% compared with other methods. These results indicate that, in such systems, the I-GCF demonstrates stronger robustness to various types of noise variations compared with other filters.

Table 3 presents the average execution time of different filters in the FM demodulation oscillation system under matched conditions. The results indicate that the I-EKF and I-CKF exhibit faster execution speed, while the computational cost of the I-UKF and I-GCF is relatively higher.

## 7. Conclusions

The proposed I-GCF algorithm, in high-dimensional and complex nonlinear environments, is able to leverage the generalized optimal conversion mechanism to remap higher-order nonlinear structures embedded in the original observations, which are difficult to be directly exploited by conventional moment approximation and local linearization methods, into decorrelated information that can be utilized for estimation update. From a physical perspective, the generalized conversion does not generate new observation information out of nothing, but rather separates effective state information that is originally compressed, aliased, or obscured in the raw observations due to nonlinear coupling induced by sensor measurements. This enables such information to be explicitly utilized by the inverse filter. For this reason, the I-GCF can achieve higher estimation accuracy and better stability in strongly nonlinear adversarial environments. In addition, this paper derives general expressions for the time complexity of both GCF and I-GCF, thereby further completing their theoretical framework.

The simulation results demonstrate that, regardless of whether the forward-filter assumption holds, the I-GCF consistently achieves higher estimation accuracy and better stability than other filters. In the Lorenz system, the estimation accuracy of the I-GCF under model-mismatch conditions even exceeds that of the I-UKF, I-CKF, and I-EKF under matched-model conditions. In the FM demodulation system, under matched-model conditions, the RMSE of the I-GCF is reduced by at least 5% compared with conventional inverse filtering methods (such as the I-UKF and I-EKF) that rely solely on moment approximation or local linearization to handle nonlinear information. However, the proposed method still has certain limitations. First, the I-GCF requires deterministic sampling in the augmented state space, and its computational complexity increases rapidly with the growth of state dimension and noise dimension. Second, the construction of the I-GCF still relies on strong model priors, including the availability of system dynamic functions, observation functions, and related noise parameters. When these priors are inaccurate in practical adversarial scenarios, or when the defender cannot precisely identify the forward filtering mechanism employed by the opponent, the performance of the algorithm may be further degraded. Therefore, future research should not only focus on reducing computational complexity, but also investigate robust and adaptive extensions of the I-GCF for high-dimensional large-scale scenarios, complex noise environments, and model-uncertain conditions.

In addition, compared with representative deep learning-enhanced filtering methods and model-driven-data-driven hybrid filtering approaches in recent years [27], such as KalmanNet [28] and its recursive extensions [29], Transformer-based state estimation methods [30], and neural network-assisted Kalman filtering methods [31], the advantage of the I-GCF lies in that it does not require training data, possesses strong theoretical interpretability, and provides a complete modeling framework and complexity analysis for inverse filtering problems in adversarial sensing. However, it still has certain limitations in terms of reliance on model priors and computational complexity. Future work will further adapt the above recent methods to inverse filtering scenarios and systematically compare their estimation accuracy, stability, robustness, and computational cost under a unified task setting, so as to more comprehensively clarify the position of the I-GCF within current research trends.

## Figures and Tables

**Table 1:** Tabular pseudo-algorithm for evaluating the implicit inverse transition ${\hat{\hat{x}}}_{k}=\tilde{f}\left(r_{k-1},P_{k-1}^{\prime },x_{k}\right)$.

Phase	Operation
**Implicit**	Start from the implicit inverse state transition ${\hat{\hat{x}}}_{k}=\tilde{f}\left(r_{k-1},P_{k-1}^{\prime },x_{k}\right)$, with $$r_{k-1}={\left[\begin{array}{cc} {\hat{\hat{x}}}_{k-1}^{T} & v_{k}^{T} \end{array}\right]}^{T}.$$
**Simulated Forward GCF**	Generate deterministic samples around $\left(r_{k-1},P_{k-1}^{\prime }\right)$: $$s_{j,k-1}=\xi_{j}\sqrt{P_{k-1}^{\prime }}+r_{k-1},\;j=1,\ldots ,N_{s}.$$ (The weight of each sample point is $\varsigma_{j}$.) Decompose each augmented sample as $${\left[\begin{array}{cc} {\left({\hat{x}}_{k-1}^{\left(j\right)}\right)}^{T} & {\left(v_{k}^{\left(j\right)}\right)}^{T} \end{array}\right]}^{T}=s_{j,k-1}.$$ For each *j*, construct the simulated opponent measurement $$z_{k}^{a}=\sum_{j=1}^{N_{s}}\varsigma_{j}\left[h\left(x_{k}\right)+v_{k}^{\left(j\right)}\right].$$ For each *j*, generate the prior sample at time *k* from ${\hat{x}}_{k-1}^{\left(j\right)}$: $${\hat{x}}_{j,k|k-1}^{\;s}=f\left({\hat{x}}_{k-1}^{\left(j\right)}\right).$$ Execute (6)–(16) using $\left({\hat{x}}_{j,k|k-1}^{\;s},z_{k}^{a}\right)$, where ${\hat{x}}_{j,k|k-1}^{\;s}$ is used as $s_{i,k|k-1}^{*}$ in those equations.
**Output**	After executing all equations, the obtained $\hat{x}$ is exactly the result of the implicit equation ${\hat{\hat{x}}}_{k}$: $${\hat{\hat{x}}}_{k}=\tilde{f}\left(r_{k-1},P_{k-1}^{\prime },x_{k}\right)\equiv {\hat{x}}_{k}.$$

**Table 2:** Execution time of different filters in the Lorenz system.

Filter	Time (ms)
I-GCF	9.270
I-CKF	1.186
I-EKF	0.009
I-UKF	0.045

**Table 3:** Execution time of different filters in the FM demodulation oscillation system.

Filter	Time (ms)
I-GCF	1.262
I-CKF	0.121
I-EKF	0.019
I-UKF	0.178

## Data Availability

The data presented in this study are available on request from the corresponding author.
